# Characterization of Hepatoma-Derived Growth Factor-Related Protein 2 Interactions with Heterochromatin

**DOI:** 10.3390/cells12020325

**Published:** 2023-01-14

**Authors:** Sarah C. Wistner, Ian A. MacDonald, Karly A. Stanley, Nathaniel A. Hathaway

**Affiliations:** 1 Center for Integrative Chemical Biology and Drug Discovery, Division of Chemical Biology and Medicinal Chemistry, UNC Eshelman School of Pharmacy, University of North Carolina at Chapel Hill, Chapel Hill, NC 27599, USA; 2 UNC Lineberger Comprehensive Cancer Center, School of Medicine, University of North Carolina at Chapel Hill, Chapel Hill, NC 27599, USA

**Keywords:** heterochromatin, HRP2, MPP8, HP1, gene regulation, chemical biology

## Abstract

The expression of genetic information is tightly controlled by chromatin regulatory proteins, including those in the heterochromatin gene repression family. Many of these regulatory proteins work together on the chromatin substrate to precisely regulate gene expression during mammalian development, giving rise to many different tissues in higher organisms from a fixed genomic template. Here we identify and characterize the interactions of two related heterochromatin regulatory proteins, heterochromatin protein 1 alpha (HP1α) and M-phase phosphoprotein 8 (MPP8), with hepatoma-derived growth factor-related protein 2 (HRP2). We find in biochemical experiments that HRP2 copurifies and co-sediments with heterochromatin-associated proteins, including HP1α and MPP8. Using the Chromatin in vivo Assay in multiple cell types, we demonstrate that HP1α-mediated gene repression dynamics are altered by the presence of HRP2. Furthermore, the knockout of HRP2 in MDA-MB-231 cells results in significant changes to chromatin structure and stability, which alter gene expression patterns. Here, we detail a mechanism by which HRP2 contributes to epigenetic transcriptional regulation through engagement with heterochromatin-associated proteins to stabilize the chromatin landscape and influence gene expression.

## 1. Introduction

The human genome catalogues a dynamic array of information. Extensive cooperation between protein macromolecules is required to precisely express pertinent genetic information in response to developmental and physiological stimuli. Careful maintenance of gene transcriptional activity—governed in part by unique patterns of DNA modifications and post-translational modifications (PTM)s on chromatin-associated histone proteins—is critical for proper cell growth and development [1]. Dysregulation of this delicately balanced system often leads to a variety of human diseases [2].

Epigenetic proteins facilitate gene regulation by modulating chromatin structure, allowing for changes in gene expression without altering the genetic code. Chromatin dynamically exists between two structural states, euchromatin and heterochromatin, which respectively enhance or occlude access to DNA by transcriptional machinery. The specific mechanisms by which facultatively silenced genes are activated upon differentiation or disease progression are not fully understood.

Until the last decade, mammalian epigenetic silencing through histone H3 lysine 9 (H3K9) methylation was thought to be directed solely by the heterochromatin protein 1 (HP1) pathway. HP1 contains several isoforms, α, β, and γ, with HP1α being the most well-characterized at heterochromatin and HP1γ often localizing to euchromatin [3,4]. Our study focuses on HP1α, a relatively small chromatin modulator, which contains a chromodomain that recognizes H3K9 di- and trimethylation (H3K9me2/3) and is linked to a chromoshadow domain by a disordered hinge region [5,6,7,8]. The chromoshadow domain is critical for HP1α engagement in protein-protein interactions with other HP1α monomers and chromatin factors, such as histone methyltransferases Suv39H1/2 and SETDB1 [9,10].

In 2015, using forward genetic screening, Tchasovnikarov and Timms et al. identified the human silencing hub (HUSH) as a second mediator of H3K9me3-mediated gene repression in humans [11]. Dependent on M-phase phosphoprotein 8 (MPP8)’s chromodomain, which selectively recognizes H3K9me3, the HUSH complex core (MPP8, TASOR, and periphilin) assembles at target loci [12]. HUSH can then recruit histone methyltransferase SETDB1 to propagate the H3K9me3 mark and induce chromatin condensation, which is often accompanied by DNA methylation [13,14,15]. Notably, this mechanism of epigenetic silencing is analogous to that seen in HP1α-mediated repression [16].

Mechanistic understanding of the events that coordinate the engagement of H3K9me3 readers with chromatin is still an active area of research. To investigate novel proteins involved in heterochromatin regulation, our lab performed a high-throughput small molecule screening in mouse embryonic stem cells (mESC)s to identify inhibitors of HP1α-mediated heterochromatin formation [17]. Subsequent chemical affinity purification and quantitative proteomics with the lead inhibitor, UNC2524, revealed the binding of known chromatin-interaction proteins, including MPP8, histone chaperone SPT6 (*Supt6H*), and transcription elongation factor LAP2 (*Tmpo*), as well as lesser characterized hepatoma-derived growth factor-related protein 2 (HRP2, *Hdgfrp2*) [18,19].

HRP2 has been associated with active gene regions, namely in the context of targeted HIV-1 integration [20,21]. HRP2 harbors a highly conserved PWWP (Pro-Trp-Trp-Pro) chromatin-binding domain, which recognizes transcriptionally accessible chromatin through association with the histone modification H3K36me3 [22]. HRP2′s H3K36 di- and trimethyl (me2/3) reader capability has been attributed to its role in transcriptional regulation, whereby HRP2 is required to promote transcriptional elongation during myoblast differentiation [23,24].

Despite strong engagement of HRP2′s PWWP domain with H3K36me2/3, Baude et al. reported that endogenous HRP2 has a high affinity for repressive histone marks, including H3K9me3 and H3K27me3, in U2OS osteosarcoma cells, suggesting that HRP2 activity is not limited to euchromatic regions and genomic location of this protein could be cell-type specific [25]. HRP2 also associates with the HUSH complex at heterochromatin in 293T human embryonic kidney cells and MDA-MB-231 triple-negative breast cancer (TNBC) cells [26]. Further, upon HRP2 knockout in LP-1 multiple myeloma cells, Wang et al. found increased levels of H3K27me3 and decreased recruitment of H3K27 demethylase, MINA [27]. These studies, in addition to our previous work, substantiate HRP2′s engagement with multiple epigenetic repressive complexes and necessitate an investigation of HRP2′s engagement with heterochromatin to advance our understanding of its role as a transcriptional regulator. Here we provide a mechanistic study examining interactions at the interface of HRP2 and H3K9me3-silenced loci.

To first validate the putative role of HRP2 in HP1α-mediated gene repression, we used shRNA technology to knockdown *Hdgfrp2* expression in mESCs, revealing that inhibition of HRP2 expression leads to disruption of HP1α activity at a GFP reporter locus. We then biochemically explored the extent to which HRP2 engages with mediators of H3K9me3-marked heterochromatin. We found that HRP2 knockout delays HP1α-mediated gene silencing and impairs the stabilization of the condensed chromatin state. Furthermore, chromatin immunoprecipitation (ChIP)-qPCR revealed that HRP2 knockout impacts H3K9me3 occupancy at *E-* and *N-cadherin*, loci which are engaged by repressive epigenetic complexes to regulate tumorigenesis in MDA-MB-231 cells [28,29]. These epigenetic changes were further reflected in significantly altered patterns of mRNA expression, demonstrating downstream transcriptional consequences of a modified chromatin landscape. Combined with our protein-protein interaction and heterochromatin kinetics studies, these data suggest that HRP2 directly influences heterochromatin assembly and disassembly to regulate gene transcription.

## 2. Materials and Methods

### 2.1. Cell Lines and Culture Conditions

The Chromatin in vivo Assay (CiA) recruitment system, in mouse embryonic stem cells (mESC)s (CiA:mESC) contains Gal4 and Zinc Finger DNA-binding arrays and a downstream nuclear eGFP gene in place of a single *Oct4* allele as previously described [30]. The CiA:mESC line containing the lentiviral construct N205 (N205: nLV EF-1α-ZFHD1-link-FKBP-HA <T2A> HP1αCS-Frbx2-V5-PGK-Blast) was used for shRNA experiments. mESCs were adapted to be grown on gelatin-coated plates without feeder cells in DMEM supplemented with 4.5 g/L glucose, 15% FBS, L-glutamate, sodium pyruvate, HEPES buffer, NEAA, 2-mercaptoethanol, LIF, and penicillin/streptomycin (mESC Media) at 37 °C with 5% CO_2_. Media was aspirated and replaced daily.

The CiA recruitment system in HEK293T (CiA:293T) cells were engineered as previously described [26]. 293T, CiA:293T, and MDA-MB-231 cells were cultured in DMEM supplemented with 4.5 g/L glucose, 10% FBS, HEPES buffer, 2-mecaptoethanol, and penicillin/streptomycin at 37 °C with 5% CO_2_. The MDA-MB-231 cell line was obtained from UNC Lineberger Comprehensive Cancer Center’s Tissue Culture Facility.

### 2.2. shRNA Construction

The doxycycline-inducible lentiviral vector pTRIPZ containing the nonsense (scramble) shRNA was used as the backbone for all subsequent shRNA cloning. The forward and reverse complement of shRNA-containing oligos (Table S1). were synthesized and slowly annealed together in an annealing buffer (10 mM Tris pH 7.5–8, 1 mM EDTA, 50 mM NaCl) to create dsDNA by raising the sample to 95 °C for 5 min and cooling the samples 1 °C/min until reaching room temperature. pTRIPZ vector and insert were digested using XhoI and EcoRI-HF (NEB, Ipswich, MA) and ligated using T4-ligase according to manufacturer instructions. Ligated constructs were transformed into One Shot Stabl3 Chemically Competent *E. coli* cells (ThermoFisher, Waltham, MA, USA) and grown on LB ampicillin agarose plates. Ligation was confirmed by DNA sequencing.

### 2.3. Lentiviral Transduction

For each viral construct to be packaged, 1.8 × 10^7^ 293T-X cells were seeded three days prior to lentiviral transduction. Two days before transduction, cells were PEI-transfected with 18 μg desired plasmid DNA construct, 4.5 μg N74 plasmid (expressing VSV-G envelope protein; 12259, Addgene, Watertown, MA, USA), 13.5 μg N75 plasmid (expressing Gag-Pol protein; 12260, Addgene,), 108 μL PEI, and 1.8 mL Opti-MEM. Sixteen hours post-transfection, toxic PEI media were removed and replaced with 25 mL of fresh media. Cells were left to produce packaged lentiviral particles for 48 h. The day prior to transduction, target cells were seeded in a 12-well format (appropriate seeding density varied by cell line). On the day of transduction, lentiviral media were carefully transferred to 50 mL conicals and centrifuged at 5000 rpm for 5 min. Without disrupting the pellet, the supernatant was removed and transferred to a 38.5 mL, open-top thin-wall ultra-clear tube, 25 × 89 mm (344058, Beckman Coulter, Brea, CA, USA). Tubes containing lentiviral media were spun at 20,000 rpm for 2.5 h at 4 °C using an SW-32 swinging rotor and buckets. The supernatant was carefully removed, and viral pellets were resuspended in 100 μL 1X PBS and added dropwise (appropriate amount of concentrated virus varied by cell line) to target cells containing 0.5 μg/mL polybrene media (Santa Cruz Biotechnology, Dallas, TX, USA). Cell plates were spun at 1000× *g* for 25 min at room temperature and then stored at 37 °C. Sixteen hours post-transduction, toxic viral media were removed and replaced with 1 mL of fresh media. Antibiotic selection began 48 h after media change.

### 2.4. shRNA Knockdown

Day 0, CiA:mESCs containing stably integrated doxycycline-inducible shRNA constructs were seeded into 96- or 24-well tissue culture plates with 10,000 or 50,000 cells per well, respectively. Day 1, media was aspirated and replaced with fresh mESC media +/− 6 nM rapamycin and +/− 1 μg/mL doxycycline to induce both csHP1α recruitment and shRNA induction as appropriate. On day 2, media was aspirated and replaced with fresh ES media +/− 6 nM rapamycin and +/− 1 μg/mL doxycycline. On day 3, media was aspirated and the cells were washed with PBS prior to cell trypsinization and sample preparation. A 96-well plate was used for flow cytometry analysis as described below. Single-cell populations were gated into GFP+ and GFP- populations. The experiment was performed in biological triplicate. Total RNA was isolated from CiA:mESCs grown in 24-well plates, extracted, and analyzed as described in “RNA Extraction and qRT-PCR.”

### 2.5. RNA Extraction and qRT-PCR

RNA was isolated in biological triplicate using RNeasy Plus Mini Kit according to the manufacturer’s protocol (74034, Qiagen, Hilden, Germany). Each RNA sample was eluted with 30 μL dH_2_O. All RNA samples were diluted by the same factor yield concentrations between 5–20 ng. RNA enrichment was quantified by qRT-PCR using Power SYBR Green RNA-to-CT 1-Step Kit (4389986, Applied Biosystems, Waltham, MA, USA) using the primers listed in Table S2. All RNA experiments were performed in biological triplicate and normalized to *β-actin* or *GAPDH*.

### 2.6. Flow Cytometry

For shRNA experiments, cell sorting was performed on a BD FACSAria II cell sorter. Upon doxycycline induction, turboRFP+ cells were sorted to enrich populations that were expressing the inducible pTRIPZ vector used for shRNA knockdown experiments. Flow cytometry was performed using an Intellicyt iQue Screener and analyzed with FlowJo software. Cell populations were gated based on forward and side scatter areas. Single-cell populations were isolated using forward scatter area by forward scatter height gating. Dead or dying cells that equally fluoresced in the GFP and APC channels upon excitation with 488 nm laser were omitted as autofluorescent. Cells were again sorted for RFP+ to include only those highly expressing shRNA constructs, and the remaining cells were gated into GFP- and GFP+ populations. Dot plots and histograms demonstrate representative samples while bar graphs and charts contain all biological replicates. Percent inhibition was calculated using the following formula: % Inhibition = [1 – (*x* − *min*)/(*max* − *min)*], where *min* = percentage of GFP+ scramble cells with CIP-HP1α recruitment, *max* = percentage of GFP+ scramble cells without CIP-HP1α recruitment, and *x* = percentage of GFP+ target cells with CIP-HP1α recruitment.

For HP1α recruitment and release time-course experiments, flow cytometry was performed using a ThermoFisher Attune NxT Flow Cytometer and analyzed with FlowJo software. Cell populations were gated based on forward and side scatter areas to identify live cells. Single-cell populations were isolated using forward scatter area by forward scatter height gating. The remaining cells were gated into GFP- and GFP+ populations. Dot plots and histograms demonstrate representative samples while bar graphs contain all biological replicates.

### 2.7. Generation and Validation of CRISPR/Cas9-Mediated HRP2 Knockout Lines

293T and CiA:293T HRP2 knockout lines were generated through transient transfection of 0.86 μg lenti-Cas9 plasmid (52962, Addgene) and 1.67 μg of *Hdgfrp2*-targeted CRISPR dual-cut gRNA-1, -2, or -3 plasmid (acquired from Transomic Technologies, Inc., Huntsville, AL, USA) or non-targeting, NT (previously published) [31]. Of note, each of these CRISPR dual-cut guides cleaves the gene-of-interest at more than one target site to increase knockout efficiency. The day prior to transfection, a 6-well plate was seeded with 5 × 10^5^ 293T or CiA:293T cells. The following day, 2.5 μg DNA (lenti-Cas9 + gRNA), 3.75 μL PEI, and 62.5 μL Opti-MEM were sterilely added to a microfuge tube, gently agitated, and incubated at room temperature for 15 min. The transfection mix was slowly dripped onto cells (one tube of transfection mix per well), and cells were stored at 37 °C. Sixteen hours after transfection, toxic PEI media were removed and replaced with fresh media. A total of 24 h after media change, 12 ng/μL blasticidin and 0.15 ng/μL puromycin were added to cells to select for those successfully transfected with lenti-Cas9 and *Hdgfrp2*-targeted gRNA, respectively. A total of 12 ng/μL blasticidin and 300 ng/μL zeocin were added to cells to select for those successfully transfected with lenti-Cas9 and NT gRNA, respectively. Approximately one week after transfection, 5 × 10^6^ of each cell type were harvested for Western blotting and densitometry analysis, whereby HRP2 protein levels were normalized to those of GAPDH and then compared to non-targeting cells to determine which *Hdgfrp2*-targeted gRNA was most efficacious at CRISPR/Cas9 gene editing. Cells from the most highly-edited *Hdgfrp2*-targeted gRNA-containing cell line were diluted into 96-well plates with a target of one cell per well to grow pure potential HRP2 knockout colonies. Wells containing zero or more than one cell were discarded. Potential HRP2 knockout colonies were then analyzed by Western blot analysis to confirm successful knockout. Successful HRP2 knockout colonies were validated by RNA extraction (see “RNA Extraction and qRT-PCR”) and immunofluorescence microscopy (see “Immunofluorescence Microscopy”).

MDA-MB-231 HRP2 knockout line was generated through stable lentiviral transduction (see “Lentiviral Transduction” above). Briefly, two days prior to transduction, 293T-X cells were PEI-transfected with lenti-Cas9, *Hdgfrp2*-targeted gRNA-3, or NT gRNA and lentiviral packaging plasmids. Toxic PEI media were removed and replaced with fresh media after 16 h. The day before transduction, a 12-well plate was seeded 1 × 10^5^ MDA-MB-231 cells per well. A total of 10 μL concentrated lenti-Cas9 virus and 20 μL concentrated gRNA virus was added to each well of MDA-MB-231 cells. A total of 48 h after transduction, cells were treated with 12 ng/μL blasticidin and 0.15 ng/μL puromycin to select for those stably transduced with lenti-Cas9 and *Hdgfrp2*-targeted gRNA, respectively. A total of 12 ng/μL blasticidin and 300 ng/μL zeocin were added to cells to select for those stably transduced with lenti-Cas9 and NT gRNA, respectively. To isolate and validate pure HRP2 knockout lines, the same procedure was used as described for 293T and CiA:293T cells. Of note, immunofluorescence microscopy was only used to validate HRP2 knockout in 293T cells.

### 2.8. Co-Immunoprecipitation and Western Blot Analysis

In microtubes, approximately 1 × 10^7^ cells per sample were harvested with 0.05% trypsin, pelleted, and washed with 1X PBS. On ice, cell pellets were resuspended in 1 mL M-PER Mammalian Protein Extraction Reagent (78501, ThermoFisher), treated with 1 μL protease inhibitors (100×) and 1 μL benzonase, and mixed by pipetting. Microtubes were incubated at 37 °C for 10 min and then inverted at room temperature for an additional 20 min. Microtubes were spun at max speed for 5 min and resulting supernatants were transferred to clean microtubes on ice. Protein concentrations were measured by Bradford assay.

Protein G Dynabeads (20 μL beads per immunoprecipitation reaction) were washed on a magnet three times with 500 μL PBST and then resuspended in 500 μL PBST. To each bead mixture, 1 μg IP primary antibody (α-HRP2 or α-HP1α) was added and inverted at room temperature for 40 min. Beads were washed on a magnet three times with 500 μL PBST then resuspended in 20 μL PBST. A total of 800 μg of protein was added to each 20 μL bead mixture to a final volume of 500 μL and inverted at 4 °C overnight.

The following day, samples were spun down gently for 30 s and placed on a magnet to remove the remaining lysate. Beads were washed on a magnet three times with wash buffer (50 mM HEPES, 1% NP-40, 150 mM NaCl in dH_2_O) then resuspended in 10.5 μL PBST. A total of 3.5 μL 4X loading dye was added to each sample (including 20 μg of whole cell lysate input), and samples were boiled at 95 °C for 5 min. Using a magnet for IP samples, all samples were loaded onto 4–20% polyacrylamide gels and run at 200 V for 40 min. Gels were transferred onto 0.45 μm PVDF membranes (IPFL00010, MilliporeSigma, Burlington, MA, USA) at 100 V for 1 h. Membranes were incubated with primary antibodies rocking overnight at 4°C, washed three times with PBST, incubated with secondary antibodies (1:10,000 in PBST, 0.1% SDS) rocking for 1 h at room temperature, then washed three times with PBST (see “Antibodies” below). Membranes were imaged using the LI-COR Odyssey.

### 2.9. Immunofluorescence Microscopy

Glass coverslips (CLS-1764-1818, Corning, Corning, NY, USA) were laid in 6-well plates and sterilized by UV light for 10 min. Coverslips were coated with 0.1% porcine gelatin for 10 min, aspirated, and allowed to air dry inside the tissue culture hood. A total of 4 × 10^5^ cells were plated in each well and allowed to settle for 48 h. Quickly after removal from the incubator, cells were fixed with 4% paraformaldehyde in 1× PBS for 10 min at room temperature. Coverslips were rinsed 3 times for 5 min each with PBST (0.1% Tween 20; P9416-100ML, Sigma-Aldrich, St. Louis, MO, USA) to remove traces of fixative. Cells were permeabilized in 0.1% Triton X-100 (X100-500ML, Sigma-Aldrich) in 1X PBS for 15–20 min while rocking. Coverslips were rinsed 3 times for 5 min each with PBST. Cells were blocked in 5% BSA + 0.25% Triton X-100 in 1X PBS for 30 min to 1 h, then the blocking buffer was aspirated. The primary antibody (see “Antibodies” below) was diluted in fresh blocking buffer, and 1 mL per well was added to cells and incubated overnight at 4 °C. One well of each cell type was omitted from this step (incubated in blocking buffer only) to control for background staining of secondary antibodies. Coverslips were rinsed 3 times for five minutes each with PBST. The secondary antibody (see “Antibodies” below) was diluted in fresh blocking buffer, and 1 mL per well was added to cells and incubated for 1 h at room temperature, protected from light. Coverslips were rinsed 3 times for 5 min each with PBST. Since the mountant (P36962, Invitrogen, Waltham, MA, USA) contained DAPI, coverslips were simultaneously counterstained and mounted onto microscope slides (12-544-2, ThermoFisher) without creating bubbles. Coverslips were sealed with clear nail polish. Microscope slides were imaged using an Olympus IX71 inverted, fluorescent microscope containing a U-HGLGPS illumination system. Images were processed using ImageJ software.

### 2.10. Sucrose Density Gradient Ultracentrifugation and Fractionation

Sucrose 20% *w/v* solution (8590-OP, Sigma-Aldrich) was diluted in sucrose solution buffer (10% 1× PBS, 0.2% 500 mM EDTA, 0.5% Triton X-100 in dH_2_O). Protease inhibitors (100X) were diluted in both 5% and 20% sucrose solutions and kept on ice. Carefully without mixing layers, each gradient was poured into the bottom of a 14 mL, polypropylene tube, 14 × 95 mm (331374, Beckman Coulter) in the following order: 2.5 mL 20% sucrose solution, 2.5 mL of 2:1 (20% to 5%) sucrose solution, 2.5 mL 1:2 (20% to 5%) sucrose solution, and 2.5 mL 5% sucrose solution. Tubes were plugged with stoppers and slowly tilted until the gradient tubes rested horizontally to diffuse for 2 min, then slowly tilted vertically to diffuse at 4 °C overnight.

An SW Ti-40 rotor and buckets were pre-chilled at 4 °C while cell lysates were prepared. Approximately 1 × 10^7^ cells were harvested per condition (non-targeting or HRP2 knockout) using 0.05% trypsin, and cell pellets were transferred to microtubes and washed with 1X PBS. An amended version of the Abcam Nuclear Fractionation Protocol was used to access nuclear lysates. Briefly, buffers A (10 mM HEPES, 1.5 mM MgCl_2_, 10 mM KCl, 0.5 mM DTT, 0.05% NP-40) and B (5 mM HEPES, 1.5 mM MgCl_2_, 0.2 mM EDTA, 0.5 mM DTT, 26% *v/v* glycerol) were prepared and stored on ice. Fresh protease inhibitors (100X) were added to buffer A before contact with cell pellets. A total of 1 mL buffer A was then added to each cell pellet, mixing thoroughly by pipetting. Microtubes were left on ice for 10 min, then centrifuged at 3000 rpm for 10 min at 4 °C. Supernatant from each microtube was removed, transferred to a clean microtube labeled “cytoplasmic extract,” and kept on ice. On ice, the pellets were resuspended in 376 μL buffer B, and 24 μL of 5 M NaCl and 1 μL benzonase were added and mixed. A total of 10 μL of each sample was set aside to check nuclei before homogenization. Samples were incubated at 37 °C for 30 min to activate the benzonase, then samples were homogenized with a Dounce homogenizer (40 strokes with pestle A, 40 strokes with pestle B). A total of 10 μL of each sample was set aside to check nuclei after homogenization. Microtubes were left on ice for 30 min and vortexed often. Nuclei samples were suspended in Trypan blue to a final concentration of 1X and loaded into microscope slides for examination. After confirmation that nuclei were successfully lysed, microtubes were centrifuged at 21,000× *g* for 20 min at 4 °C. Supernatant from each microtube was removed, transferred to a clean microtube labeled “nuclear extract,” and kept on ice.

Thyroglobulin (200 μL of 20 mg/mL thyroglobulin in 50 mM Tris-HCl pH 7.5, 100 mM KCl, and dH_2_O) and Precision-Plus (10 μL Precision-Plus Protein Standards in 190 μL sucrose buffer solution) standards were prepared on ice. Bradford assay was used to determine the protein concentrations of each cytoplasmic and nuclear extract sample. Carefully without disrupting gradient, 1.3 mg of nuclear extract or protein standard (to an equal final volume for all samples and standards to avoid non-experimental differences in gradient migration) was loaded on top of the gradient.

Gradient tubes (unplugged) were loaded into the SW Ti-40 buckets and sealed. If necessary, a small amount of sucrose buffer solution was added to the top of a gradient to balance the buckets. Buckets were then loaded onto the rotor in the ultracentrifuge and spun at 250,000× *g* for 16 h at 4 °C. Microtubes for fraction collecting were pre-labeled 1–21 and set on ice. Samples were removed from ultracentrifuge without disrupting gradients. One gradient at a time was kept out for fractionation, and all other gradient tubes were kept at 4 °C. Using a p1000, carefully removed 500 μL from the top of the gradient without dipping below the meniscus and without expelling any sample to avoid mixing the gradient. Fraction 1 was transferred to its respective microtube on ice. Fractionation was repeated until the entire gradient was removed from the gradient tube and then until each gradient tube was fractionated. A total of 10.5 μL of each fraction from all samples were transferred to clean microtubes on ice for Western blot analysis or SYPRO Ruby staining. The remaining samples were stored at −80 °C. A total of 3.5 μL of 4X loading dye was added to each 10.5 μL fraction (including 10 μg of cytoplasmic and nuclear inputs), and samples were boiled at 95 °C for 5 min. Inputs and fractions were loaded onto 4–20% polyacrylamide gels and run at 200 V for 40 min.

Gels containing protein standards were transferred into 20 mL fixed solution (50% methanol, 7% acetic acid) and incubated with rocking for 30 min at room temperature. Disposed of fix buffer and repeated with fresh fix buffer. Disposed of fixed buffer and incubated with 40 mL SYPRO Ruby Protein Gel Stain (ThermoFisher) with rocking overnight at room temperature. Disposed of staining buffer, added 20 mL wash buffer (10% methanol, 7% acetic acid), and incubated with rocking for 15 min at room temperature. Disposed of wash buffer and repeated with fresh wash buffer. Disposed of wash buffer, added 20 mL dH_2_O, and incubated with rocking for 5 min at room temperature. Gels were imaged using an E-Gel Imager (Life Technologies, Carlsbad, CA, USA).

Gels containing nuclear extract fractions were transferred onto 0.45 μm PVDF membranes as described above under “Co-Immunoprecipitation and Western Blot Analysis” and incubated with primary antibodies against HRP2, HP1α, MPP8, GAPDH, and BRD4 (see “Antibodies” below). Following incubation with secondary antibodies, membranes were imaged using the LI-COR Odyssey and aligned in order of fraction for migration analysis.

### 2.11. Chromatin Immunoprecipitation (ChIP) and qPCR

ChIP was performed as previously described in Waybright et al. 2021 with minor adjustments [26]. Briefly, 16 × 10^6^ MDA-MB-231 non-targeting (HRP2^WT^) or HRP2 knockout (HRP2^KO^) cells were harvested in biological duplicate to allow for 3.2 × 10^6^ cells per antibody during the immunoprecipitation (IP) step. Cells were formaldehyde crosslinked, nuclei were isolated, and chromatin was sonicated to 200–500 bp fragments. Inputs were purified using a Qiagen MinElute PCR Purification Kit while the remaining chromatin was immunoprecipitated with 2 μg antibody (α-IgG or α-H3K9me3) using 20 μL Protein G Dynabeads (ThermoFisher) per IP sample. IP samples were purified using a Qiagen MinElute PCR Purification Kit, and samples were analyzed using the percent input method following qPCR (QuantStudio ViiA 7 System). Primers are listed in Table S3.

### 2.12. Antibodies

The following primary antibodies were used for immunoblot analysis: α-HRP2 (0.5 μg/mL; 15134-1-AP, Proteintech, Rosemont, IL, USA), α-HP1α (2 μg/mL; bsm-33377M, Bioss, Woburn, MA, USA), α-MPP8 (1:1000; 16796-1-AP, Proteintech), α-H3K9me3 (1:1000; ab8898, Abcam, Cambridge, United Kingdom), α-GAPDH (1:2000; ab8245, Abcam), α-BRD4 (1:5000; ab128874, Abcam), α-TASOR (1:500; HPA006735, Atlas, Bromma, Sweden).

The following secondary antibodies were used for immunoblot analysis: IRDye 800CW Goat anti-Rabbit IgG (1:10,000; 925-32211, LI-COR, Lincoln, NE, USA), IRDye 680RD Goat anti-Mouse IgG (1:10,000; 925-68070, LI-COR).

The following antibodies were used for immunofluorescence staining: α-HRP2 (1:1000; 15134-1-AP, Proteintech), α-β actin (1:200; ab8226, Abcam), Goat anti-Rabbit IgG AlexaFluor 488 (1 μg/mL; ab150077, Abcam), Goat anti-Mouse IgG AlexaFluor 568 (1 μg/mL; A11004, ThermoFisher).

The following antibodies were used for ChIP at 2 μg per IP: α-IgG (011-000-003, Jackson ImmunoResearch, West Grove, PA, USA), α-H3K9me3 (ab8898, Abcam).

## 3. Results

### 3.1. Hdgfrp2 Knockdown Inhibits HP1α-Mediated Gene Repression

**Figure 1:**
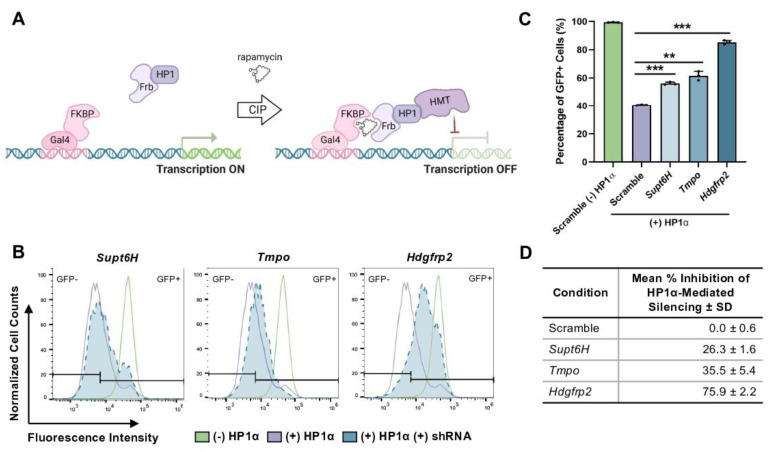
*Hdgfrp2* knockdown inhibits HP1α-mediated gene repression. (**A**) Schematic overview of the Chromatin in vivo Assay, or CiA system. A Gal4 binding site lies just upstream of the GFP reporter, which allows Gal4-FKBP to be recruited to the reporter locus. The addition of rapamycin initiates CIP between Frb-csHP1α and Gal4-FKBP, resulting in HP1α-mediated GFP repression. (**B**) CiA:mESCs containing inducible shRNA constructs targeted against *Supt6H*, *Tmpo*, and *Hdgfrp2* were induced with 1 μg/mL doxycycline for 48 hrs +/− 6 nM rapamycin. Flow cytometry histograms represent fluorescence intensity levels of shRNA-targeted cells (blue) during active CIP-HP1α recruitment compared to nonsense (scramble) shRNA control cells with (purple) and without (green) CIP-HP1α recruitment. (**C**) Mean percentages of GFP+ cells after 48 h of treatment with doxycycline: Scramble (-) HP1α = 99.4 ± 0.2; Scramble (+) HP1α = 40.7 ± 0.4; *Supt6H* (+) HP1α = 56.1 ± 1.0; *Tmpo* (+) HP1α = 61.5 ± 3.2; *Hdgfrp2* (+) HP1α = 85.2 ± 1.3. Statistical significance was calculated using an unpaired *t*-test (*n* = 3; ** *p* ≤ 0.01, *** *p* ≤ 0.001). (**D**) Mean % inhibition of HP1α-mediated silencing.

To validate our pursuit of HRP2 as a putative regulator of H3K9me3-mediated heterochromatin, we utilized the Chromatin in vivo Assay (CiA) to perturb chromatin dynamics in real time [30]. In mESCs, the CiA system (CiA:mESC) uses chemically-induced proximity, or CIP, to recruit epigenetic modifiers to an *Oct4*/GFP reporter gene. In mESCs, haplosufficient *Oct4* is highly expressed to maintain cell stemness prior to differentiation, after which it is rapidly repressed by the HP1α pathway [32]. Just upstream of the *Oct4*/GFP locus lies Gal4- and Zinc Finger-DNA binding arrays. Through stable expression of a Gal4-DNA binding domain fused to FK506-binding protein (Gal4-FKBP) and an FKBP-rapamycin binding domain fused to the chromoshadow domain of HP1α (Frb-csHP1α), the addition of 6 nM rapamycin to CiA:mESCs allows CIP-mediated recruitment of HP1α-associated epigenetic silencing machinery (Figure 1A). Induced *Oct4*/GFP silencing mimics that which occurs naturally during stem cell differentiation, yet it can be quantified in individual cells over time using flow cytometry.

With temporal control over HP1α-induced *Oct4*/GFP silencing, we utilized inducible shRNA knockdown directed against *Hdgfrp2*, as well as other top UNC2524 targets, *Supt6H* and *Tmpo*, to determine whether inhibiting any of these putative binding partners would lead to interference of HP1α-mediated repression, recapitulating the compound’s inhibitory phenotype. Lentiviral transduction of CiA:mESCs with the pTRIPZ vector allowed for stable integration of the shRNA construct under a doxycycline inducible promoter. Of note, a single CiA vector (N205: ZFHD1-FKBP T2A cleavage site Frb-csHP1α) was used for this study to allow for the selection of the shRNA while maintaining the capacity to recruit HP1α to the *Oct4*/GFP locus. CiA:mESCs expressing the shRNA constructs were grown with rapamycin in the presence of 1 μg/mL doxyclycine for 48 h. Knockdown of target mRNA levels was confirmed by qRT-PCR and normalized to *β-actin* (Figure S1). *Supt6H* was suppressed by ~40% while *Tmpo* and *Hdgfrp2* were knocked down by 70–80%.

Cells were subsequently analyzed by flow cytometry to determine if the shRNA knockdown of target genes could recapitulate the inhibition of HP1α-mediated GFP repression when compared to a nonsense shRNA control (scramble) (Figure 1B). During active CIP-HP1α recruitment, we found that *Tmpo* and *Hdgfrp2* knockdown resulted in the largest percentage of cells that remained GFP positive relative to HP1α recruitment in the absence of shRNA induction (Figure 1C and Figure S2B). Comparing these data to the percentage of GFP-positive (GFP+) scramble cells with (min. inhibition) and without (max. inhibition) CIP-HP1α recruitment revealed that *Supt6H* and *Tmpo* displayed mild inhibitory effects (25–35%) while *Hdgfrp2* inhibited *Oct4*/GFP repression by more than 75% (Figure 1D). In agreement with recent literature, our data suggest that HRP2 significantly impacts the epigenetic regulation of gene transcription [23,24,27]. We next focused on the mechanistic role of HRP2 in regulating the H3K9me3-decorated heterochromatin landscape and gene expression.

### 3.2. HRP2 Interacts and Co-Migrates with H3K9me3-Depositing Complexes

**Figure 2:**
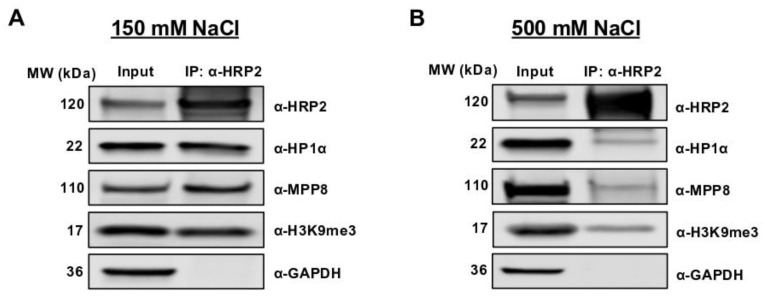
HRP2 stably interacts with HP1α, MPP8, and H3K9me3 at increasing salt concentrations. (**A**) HRP2 strongly co-immunoprecipitates with HP1α, MPP8, and H3K9me3, and does not interact with GAPDH (negative control) at 150 mM NaCl. (**B**) HRP2 maintains association with HP1α, MPP8, and H3K9me3 but does not interact with GAPDH (negative control) at 500 mM NaCl.

To explore the functionality of HRP2 at heterochromatin in other mammalian cell types such as human cancer cell lines, we first performed co-immunoprecipitations in HEK 293T cells. In congruence with previous studies in human lines, we found that H3K9me3 immunoprecipitated strongly with HRP2 (Figure 2A and Figure S3) [25]. We also confirmed that HRP2 stably interacts with HP1α and MPP8 at increasing salt concentrations, suggesting that at least some of HRP2′s affinity for H3K9me3 could be attributed to potential complex formation with HP1α and/or HUSH (Figure 2A,B and Figure S3). To determine whether HRP2 is integrated into higher molecular weight repressive complexes, we performed sucrose density gradient ultracentrifugation in the presence and absence of HRP2, followed by immunoblot analysis.

**Figure 3:**
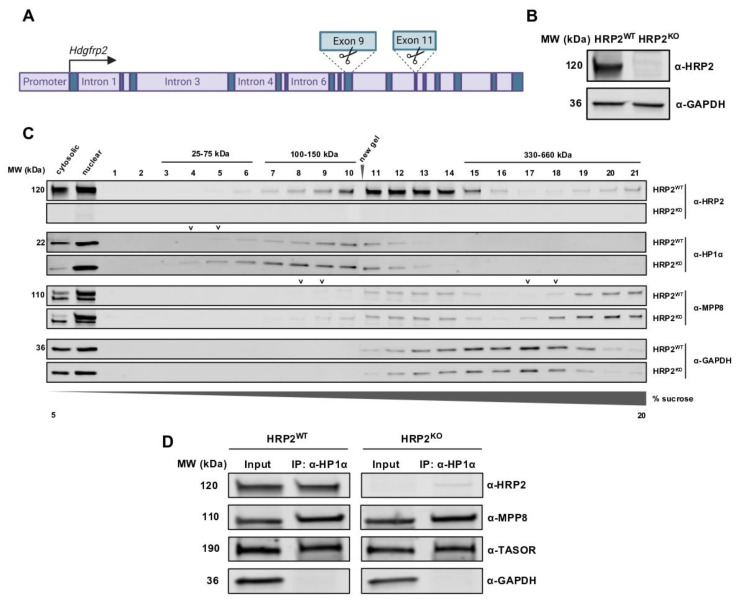
HRP2 co-sediments with H3K9me3-depositing complexes, and loss of HRP2 do not weaken HP1α-HUSH interactions. (**A**) Map of CRISPR/Cas9 dual cutter gRNA target sites along the *Hdgfrp2* gene. (**B**) Immunoblot of HRP2^WT^ and HRP2^KO^ 293T whole-cell lysates comparing levels of HRP2 and GAPDH. (**C**) Immunoblot detecting putative molecular complexes associated with HRP2, resulting from density gradient ultracentrifugation and fractionation. A total of 1.3 mg of HRP2^WT^ or HRP2^KO^ 293T nuclear lysate was run on a 5–20% sucrose gradient. The resulting gradient was fractionated in 500 μL aliquots from lowest density (1) to highest density (21). Fractions distinguished with “**˅**” indicate leftward shifts of proteins to lower density fractions in the absence of HRP2 expression compared to wild-type conditions. (**D**) HP1α co-immunoprecipitates with HUSH components in HRP2^WT^ and HRP2^KO^ 293T whole-cell lysates.

To generate an HRP2 knockout (HRP2^KO^) line in 293Ts, cells were transfected with both CRISPR/Cas9 and an *Hdgfrp2*-targeted CRISPR dual-cut guide RNAs (gRNA)s designed to create two double-stranded breaks in the *Hdgfrp2* genomic sequence (Figure S4A). After determining the most efficacious HRP2-targeted CRISPR dual-cutter sgRNA, cells from the gRNA-3 population were plated for single colony selection to isolate a genetically pure knockout population (Figure 3A,B and Figure S4B,C). HRP2 knockout (HRP2^KO^) was confirmed via immunoblot analysis, mRNA extraction and qRT-PCR at gene regions both upstream and downstream of the double-stranded breaks, as well as immunofluorescence microscopy (Figure S5). A non-targeting single gRNA was used to generate an HRP2 wildtype control cell line (HRP2^WT^) and account for the presence of CRISPR/Cas9.

To analyze the consequence of HRP2 knockout on H3K9me3-depositing complex composition, we used a density gradient ultracentrifugation protocol adapted from Lu et al. and Fernandez-Martinez et al. We extracted nuclear lysates from HRP2^WT^ and HRP2^KO^ cells and loaded 1.3 mg of protein onto 5–20% continuous sucrose gradients for ultracentrifugation, by which endogenous monomeric proteins and multi-protein complexes were separated by molecular weight [33,34]. The resulting distribution of proteins was fractionated in aliquots of 500 μL each and analyzed by immunoblot (Figure 3C).

After aligning and comparing HRP2^WT^ gradient fractions against several antibodies, we found that HRP2 (120 kDa) co-migrates with HP1α (22 kDa) at lower density fractions in agreement with molecular standards ranging from 100–150 kDa in size and with MPP8 (110 kDa) at higher density fractions (Figure S6). To investigate the likelihood that these instances of co-sedimentation represent the isolation of HRP2-comprised protein complexes, we compared HRP2^WT^ gradient fractions to those of HRP2^KO^. For fractions in which we observed co-sedimentation of HRP2 with HP1α or MPP8 in HRP2^WT^ cells, we detected leftward shifts to lower densities in the immunoblot bands representing HP1α- and MPP8-associated complexes in HRP2^KO^ cells, confirming a loss in each complex’s molecular mass in the absence of HRP2. These shifts were not observed for control proteins GAPDH, a non-epigenetic protein, or BRD4, and epigenetic activator (Figure 3C and Figure S7) [35].

Interestingly, HRP2 co-migrates with MPP8 in two distinct collections of fractions in the 200–300 kDa range, likely indicative of HRP2 associating with monomeric MPP8, and in the 500–600 kDa range. Given the recent confirmation that periphilin (53 kDa) dimerization is required for HUSH-driven epigenetic silencing, these higher molecular weight complexes may reflect HRP2 association with HUSH-bound MPP8 [36]. Unfortunately, we were unable to capture the migration of TASOR (189 kDa) at its full molecular weight—despite its detection in our nuclear lysate input—likely due to observed potential protein fragmentation within the gradient (Figure S8).

We also observed instances of HRP2 co-migration with both HP1α and MPP8 within the same fractions, consistent with previously reported associations of HUSH with all HP1 isoforms [26]. Interestingly, this putative complex did not dissociate in the absence of HRP2, suggesting that HRP2 is not required for all HP1α-HUSH interactions. Co-immunoprecipitation analysis confirmed that HP1α association with MPP8 or TASOR is not dependent on HRP2 expression (Figure 3D and Figure S9).

### 3.3. Loss of HRP2 Disrupts HP1α-Mediated Heterochromatin Stability

Given that HRP2 associates strongly with HP1α and other H3K9me3-depositing molecular players, we were interested in investigating the impact of HRP2 on heterochromatin dynamics. We used a previously described variation of our CiA system in 293T cells (CiA:293T), in which a GFP reporter is located downstream of Gal4- and Zinc Finger-binding arrays [26]. By fusing a Gal4 DNA-binding domain (Gal4-DBD) to the C-terminus of HRP2 (HRP2-Gal4), we were able to directly recruit HRP2 to the GFP reporter and observe changes in expression. Using Gal4-DBD only (no tethered repressor) as a positive control for GFP expression and Gal4-DBD fused to the N-terminus of either HP1α’s chromoshadow domain (Gal4-csHP1α) or MPP8 (Gal4-MPP8) as negative controls, we found that HRP2 induces slight but not complete gene silencing (Figure S10). Because HRP2 alone was incapable of initiating full epigenetic silencing, we hypothesized that the role of HRP2 in H3K9me3-mediated gene repression is not direct.

**Figure 4:**
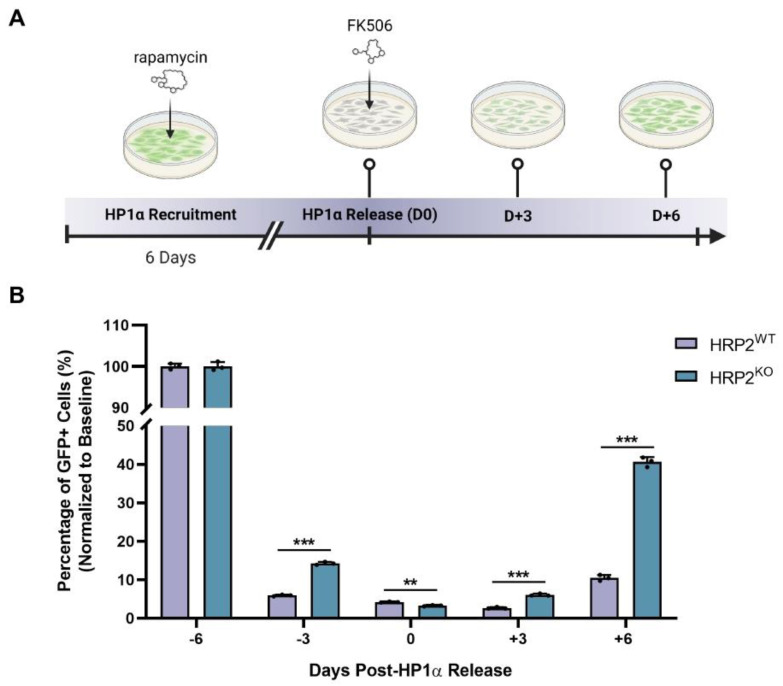
Loss of HRP2 disrupts HP1α-mediated heterochromatin stability. (**A**) Schematic of time course experiment whereby HP1α was recruited to a GFP reporter locus in CiA:293T cells during 6 days of 3 nM rapamycin treatment, then released from the reporter through competitive inhibition of rapamycin with 100 nM FK506 over 6 days. Flow cytometry was used to measure fluorescence intensities at Day −6 (D-6: pre-HP1α recruitment), Day −3 (D-3: mid-HP1α recruitment), Day 0 (D0: HP1α release), Day +3 (D + 3: post-HP1α release), and Day +6 (D + 6: post-HP1α release). (**B**) Mean percentages of GFP+ cells during 6 days of HP1α recruitment followed by 6 days of HP1α release: Day −6 HRP2^WT^ = 100.0 ± 0.7; Day −6 HRP2^KO^ = 100 ± 1.0; Day −3 HRP2^WT^ = 5.9 ± 0.2; Day −3 HRP2^KO^ = 14.3 ± 0.4; Day 0 HRP2^WT^ = 4.2 ± 0.1; Day 0 HRP2^KO^ = 3.3 ± 0.2; Day +3 HRP2^WT^ = 2.7 ± 0.2; Day +3 HRP2^KO^ = 6.1 ± 0.3; Day +6 HRP2^WT^ = 10.5 ± 0.8; Day +6 HRP2^KO^ = 40.6 ± 1.3. Cells were gated into GFP+ populations based on the maximal GFP expression level of each cell line at D-6 prior to HP1α recruitment (baseline). Statistical significance was calculated using an unpaired *t*-test (*n* = 3; ** *p* ≤ 0.01, *** *p* ≤ 0.001).

To further probe the role of HRP2 in HP1α-driven heterochromatin formation, we generated HRP2 knockout (HRP2^KO^) and non-targeting (HRP2^WT^) variants of our CiA:293T reporter line (Figure S11). Similar to our CiA:mESC system, CiA:293T cells were stably transduced with both Gal4-FKBP and Frb-csHP1α prior to CRISPR/Cas9 editing to allow rapamycin-inducible recruitment of HP1α to the GFP reporter and real-time analysis of HP1α pathway activity. Importantly, HP1α can be released from the reporter locus with the addition of 100 nM FK506, which competitively inhibits the binding of rapamycin to FKBP and reverses HP1α binding [37]. To capture the impact of HRP2 loss on HP1α-mediated heterochromatin assembly and disassembly, we used flow cytometry to assess GFP expression in HRP2^WT^ and HRP2^KO^ cells prior to HP1α recruitment (D-6, baseline), mid-HP1α recruitment (D-3), initial HP1α release (D0), and post-HP1α release (D + 3 and D + 6) (Figure 4A). By comparing the percentage of GFP+ cells at each time point, we found that HRP2 knockout significantly delays HP1α-mediated silencing of the reporter locus (Figure 4B and Figure S12). Notably, these data recapitulate the effect of *Hdgfrp2* shRNA knockdown on *Oct4*/GFP silencing in CiA:mESCs at the early time sampling (Figure 1B,D). We also observed significantly decreased retention of the repressive phenotype, suggesting that HRP2 contributes to heterochromatin stability.

### 3.4. HRP2 Knockout Impacts H3K9me3 Occupancy at E- and N-Cadherin, Resulting in Altered Transcriptional Activity

**Figure 5:**
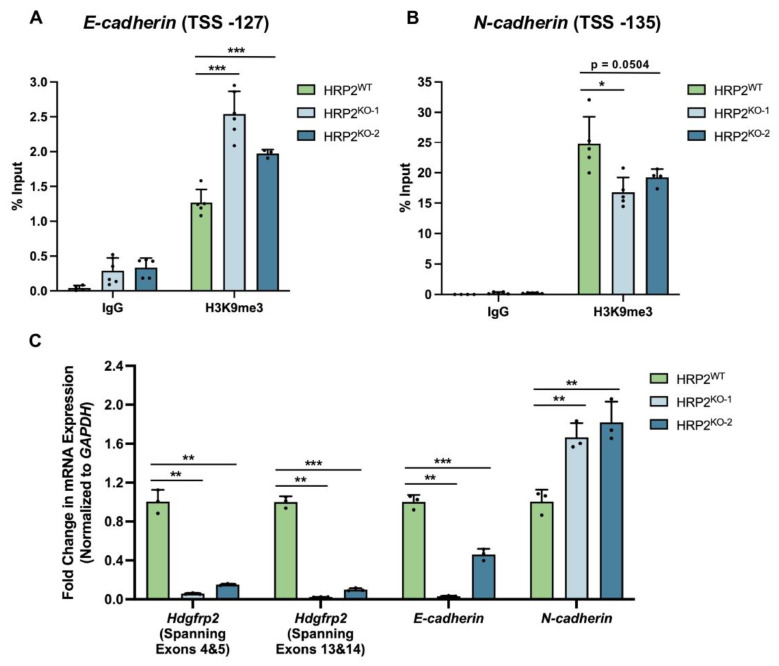
HRP2 knockout in MDA-MB-231 cells impacts H3K9me3 occupancy at *E-* and *N- cadherin*, resulting in altered transcriptional activity. (**A**) ChIP-qPCR analysis comparing enrichment of H3K9me3 at the *E-cadherin* promoter in HRP2 wildtype and knockout cells. H3K9me3 enrichment levels, measured as a percentage of input, are as follows: HRP2^WT^ = 1.3 ± 0.2; HRP2^KO−1^ = 2.5 ± 0.3; HRP2^KO−2^ = 2.0 ± 0.1. (**B**) ChIP-qPCR analysis comparing enrichment of H3K9me3 at the *N-cadherin* promoter in HRP2 wildtype and knockout cells. H3K9me3 enrichment levels, measured as a percentage of input: HRP2^WT^ = 24.7 ± 4.5; HRP2^KO−1^ = 16.8 ± 2.5; HRP2^KO−2^ = 19.2 ± 1.4. TSS indicates the position (in bp) from the transcriptional start site. (**C**) Analysis of *Hdgfrp2*, *E-cadherin*, and *N-cadherin* mRNA expression changes under HRP2 knockout conditions. Fold-change in *Hdgfrp2* expression (spanning exons 4 and 5): HRP2^WT^ = 1.00 ± 0.12; HRP2^KO−1^ = 0.06 ± 0.01; HRP2^KO−2^ = 0.15 ± 0.01. Fold-change in *Hdgfrp2* expression (spanning exons 13 and 14): HRP2^WT^ = 1.001 ± 0.058; HRP2^KO−1^ = 0.023 ± 0.002; HRP2^KO−2^ = 0.098 ± 0.015. Fold-change in *E-cadherin* expression: HRP2^WT^ = 1.002 ± 0.072; HRP2^KO−1^ = 0.031 ± 0.005; HRP2^KO−2^ = 0.461 ± 0.059. Fold-change in *N-cadherin* expression: HRP2^WT^ = 1.0 ± 0.1; HRP2^KO−1^ = 1.7 ± 0.1; HRP2^KO−2^ = 1.8 ± 0.2. Statistical significance was calculated using an unpaired *t*-test (*n* ≥ 3; * *p* ≤ 0.05, ** *p* ≤ 0.01, *** *p* ≤ 0.001).

To confirm the mechanism by which HRP2 knockout influences transcriptional activity, we used an endogenous approach to analyze the chromatin landscape at a well-characterized gene locus, *E-cadherin*, in a triple-negative breast cancer cell (TNBC) line, MDA-MB-231. *E-cadherin* is epigenetically silenced by H3K9me3 installation and DNA methylation to facilitate the epithelial-to-mesenchymal transition (EMT) in metastatic TNBC cells [28,38]. It has previously been shown that HRP2 co-localizes with the HUSH complex at this locus [26]. After generating two clonal HRP2 knockout lines in MDA-MB-231 cells (HRP2^KO−1^ and HRP2^KO−2^), we used ChIP to examine whether HRP2 influences H3K9me3 enrichment at a non-engineered, heterochromatinized locus (Figure S13). Interestingly, loss of HRP2 significantly increased H3K9me3 levels at the *E-cadherin* promoter region (TSS -127), resulting in decreased mRNA expression (Figure 5A,C).

To determine whether HRP2 knockout results in decreased transcription, we wanted to examine another gene. We next investigated H3K9me3 occupancy at *N-cadherin*, a locus whose expression is negatively correlated with *E-cadherin* in wild-type MDA-MB-231 cells [39]. At the *N-cadherin* promoter region (TSS -135), we observed significantly decreased enrichment of H3K9me3 in both HRP2^KO^ lines (Figure 5B). Changes to the epigenetic landscape were reflected in increased levels of *N-cadherin* mRNA expression (Figure 5C). It is important to note that while *E-cadherin* has been previously characterized as an H3K9me3-targeted locus in MDA-MB-231 cells, changes to chromatin structure and gene expression at *N-cadherin* could be a subsidiary effect of HRP2 loss. Even so, these data suggest that HRP2 is critical for maintaining established transcriptional patterns, such as those involved in EMT. Interestingly, HRP2 knockout also induced consistent changes to MDA-MB-231 cell morphology, including increased size, loss of membrane boundaries, enlarged nuclei, and increased granularity (Figure S14). Together with our density gradient characterization and heterochromatin kinetics studies, our findings support a mechanism for HRP2-mediated transcriptional regulation, whereby HRP2 works in complex with repressive epigenetic machinery to stabilize regions of H3K9me3-marked heterochromatin.

## 4. Discussion

Taken together, our work suggests that HRP2 influences heterochromatin assembly and disassembly to effectively regulate transcription. Others have demonstrated that HRP2 is required for the activation of precise gene expression patterns during cellular differentiation and disease [23,24,27]. While these studies highlight the H3K36me2/3-targeted activity of HRP2, the PWWP motif conserved within the HDGF family has documented promiscuity for a variety of methyl marks and DNA substrates [22,40,41,42]. We and others have shown that HRP2 also binds tightly to transcriptionally silent marks [25,27]. Not uncommon for epigenetic modulators, such as closely-associated HP1 proteins, it appears the activity and distribution of HRP2 are not limited to a single chromatin state [4,43,44].

Given that HRP2 interacted with our HP1 pathway inhibitor in previous work, we became interested in understanding how HRP2 engages with heterochromatin-associated proteins to influence transcriptional outcomes [17]. Here we detailed interactions between HRP2 and two critical heterochromatin regulatory proteins. In an environment where HP1α recruitment is chemically induced, loss of HRP2 results in the inhibition of H3K9me3-driven heterochromatin formation. We found that disruption of HRP2 not only alters gene expression, but also affects dynamic response rates to HP1α-mediated changes in chromatin structure. In a more endogenous cellular environment, HRP2 knockout drives quantifiable changes to the H3K9me3 chromatin signature, specifically at loci involved in EMT, ultimately altering gene transcription and cell morphology. An important limitation of our work is the characterization of HRP2 activity at exogenous reporter loci and two select endogenous genes. Future genome-wide analyses will be informative to reveal the global patterns that HRP2 plays in regulating the epigenomic landscape.

**Figure 6:**
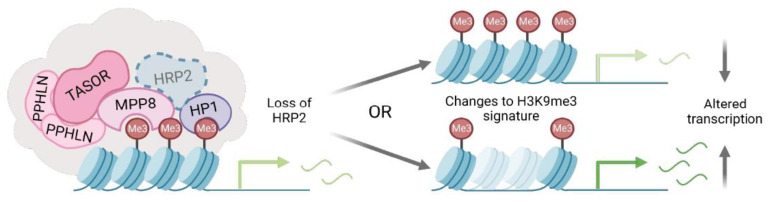
Illustration of HRP2 interacting with heterochromatin regulatory proteins, HP1 and MPP8, to maintain H3K9me3-marked chromatin signatures. Disruption of HRP2 results in changes to heterochromatin dynamics and altered transcriptional outcomes.

Together with previous studies, our data suggest that HRP2 is important for maintaining a balance between gene transcription and repressive elements. We found that while HRP2 may contribute to *de novo* heterochromatin formation, its interactions with repressive epigenetic complexes predominantly support the stabilization of H3K9me3-marked heterochromatin domains within the existing chromatin landscape (Figure 6). It will be exciting in future studies to examine other loci in different cellular contexts to further explore the role of HRP2 in maintaining chromatin states.

## Data Availability

All data are presented in the primary figures or in the supplemental information. Any raw data are available upon request.

## Supplementary Materials

The following supporting information can be downloaded at: https://www.mdpi.com/article/10.3390/cells12020325/s1, Figure S1: qRT-PCR confirms knockdown of shRNA-targeted genes; Figure S2: *Supt6H*, *Tmpo*, and *Hdgfrp2* shRNA knockdowns inhibit HP1α-mediated gene silencing to varying degrees; Figure S3: Full immunoblots corresponding to Figure 2; Figure S4: Determination of *Hdgfrp2*-targeted gRNA efficacy; Figure S5: Validation of *Hdgfrp2*-targeted CRISPR/Cas9 knockout in 293T cells; Figure S6: Sucrose gradient ultracentrifugation and fractionation of protein standards; Figure S7: Biological repeats of sucrose gradient fractions; Figure S8: Incomplete capture of TASOR migration within sucrose gradient fractions; Figure S9: Full immunoblots corresponding to Figure 3D; Figure S10: HRP2 direct recruitment induces slight but not complete gene silencing; Figure S11: Validation of *Hdgfrp2*-targeted CRISPR/Cas9 knockout in CiA:293T cells; Figure S12: Flow cytometry gating used to compare HRP2WT and HRP2KO GFP+ populations over time; Figure S13: Validation of *Hdgfrp2*-targeted CRISPR/Cas9 knockout in MDA-MB-231 cells; Figure S14: HRP2 knockout induces changes in MDA-MB-231 cellular morphology, including increased size, loss of membrane boundaries, enlarged nuclei, and increased granularity; Table S1: shRNA Sequences; Table S2: RNA qRT-PCR Primer Sequences; Table S3: ChIP Primer Sequences.

## References

[1] Jenuwein T., Allis C.D. (2001). Translating the histone code. Science.

[2] Pfister S.X., Ashworth A. (2017). Marked for death: Targeting epigenetic changes in cancer. Nat. Rev. Drug Discov..

[3] Minc E., Allory Y., Worman H.J., Courvalin J.C., Buendia B. (1999). Localization and phosphorylation of HP1 proteins during the cell cycle in mammalian cells. Chromosoma.

[4] Vakoc C.R., Mandat S.A., Olenchock B.A., Blobel G.A. (2005). Histone H3 lysine 9 methylation and HP1γ are associated with transcription elongation through mammalian chromatin. Mol. Cell.

[5] Jacobs S.A., Taverna S.D., Zhang Y., Briggs S.D., Li J., Eissenberg J.C., Allis C.D., Khorasanizadeh S. (2001). Specificity of the HP1 chromo domain for the methylated N-terminus of histone H3. EMBO J..

[6] Nielsen P.R., Nietlispach D., Mott H.R., Callaghan J., Bannister A., Kouzarides T., Murzin A.G., Murzina N.V., Laue E.D. (2002). Structure of the HP1 chromodomain bound to histone H3 methylated at lysine 9. Nature.

[7] Smothers J.F., Henikoff S. (2001). The Hinge and Chromo Shadow Domain Impart Distinct Targeting of HP1-Like Proteins. Mol. Cell. Biol..

[8] Kilic S., Bachmann A.L., Bryan L.C., Fierz B. (2015). Multivalency governs HP1α association dynamics with the silent chromatin state. Nat. Commun..

[9] Peters A.H.F.M., Mermoud J.E., O’carroll D., Pagani M., Schweizer D., Brockdorff N., Jenuwein T. (2002). Histone H3 lysine 9 methylation is an epigenetic imprint of facultative heterochromatin. Nat. Genet..

[10] Schultz D.C., Ayyanathan K., Negorev D., Maul G.G., Rauscher F.J. (2002). SETDB1: A novel KAP-1-associated histone H3, lysine 9-specific methyltransferase that contributes to HP1-mediated silencing of euchromatic genes by KRAB zinc-finger proteins. Genes Dev..

[11] Tchasovnikarova I.A., Timms R.T., Matheson N.J., Wals K., Antrobus R., Göttgens B., Dougan G., Dawson M.A., Lehner P.J. (2015). Epigenetic silencing by the HUSH complex mediates position-effect variegation in human cells. Science.

[12] Li J., Li Z., Ruan J., Xu C., Tong Y., Pan P.W., Tempel W., Crombet L., Min J., Zang J. (2011). Structural basis for specific binding of human MPP8 chromodomain to histone H3 methylated at lysine 9. PLoS ONE.

[13] Chang Y., Sun L., Kokura K., Horton J.R., Fukuda M., Espejo A., Izumi V., Koomen J.M., Bedford M.T., Zhang X. (2011). MPP8 mediates the interactions between DNA methyltransferase Dnmt3a and H3K9 methyltransferase GLP/G9a. Nat. Commun..

[14] Cruz-Tapias P., Robin P., Pontis J., Del Maestro L., Ait-Si-Ali S. (2019). The H3K9 Methylation Writer SETDB1 and its Reader MPP8 Cooperate to Silence Satellite DNA Repeats in Mouse Embryonic Stem Cells. Genes.

[15] Müller I., Moroni A.S., Shlyueva D., Sahadevan S., Schoof E.M., Radzisheuskaya A., Højfeldt J.W., Tatar T., Koche R.P., Huang C. (2021). MPP8 is essential for sustaining self-renewal of ground-state pluripotent stem cells. Nat. Commun..

[16] Van Lint C. (2018). Stop HUSHing on SIV/HIV. Nat. Microbiol..

[17] MacDonald I.A., Butler K.V., Herring L.E., Clinkscales S.E., Yelagandula R., Stecher K., Bell O., Graves L.M., Jin J., Hathaway N.A. (2019). Pathway-Based High-Throughput Chemical Screen Identifies Compounds That Decouple Heterochromatin Transformations. SLAS Discov..

[18] Wang A.H., Zare H., Mousavi K., Wang C., Moravec C.E., Sirotkin H.I., Ge K., Gutierrez-Cruz G., Sartorelli V. (2013). The histone chaperone Spt6 coordinates histone H3K27 demethylation and myogenesis. EMBO J..

[19] Narain A., Bhandare P., Adhikari B., Backes S., Eilers M., Dölken L., Schlosser A., Erhard F., Baluapuri A., Wolf E. (2021). Targeted protein degradation reveals a direct role of SPT6 in RNAPII elongation and termination. Mol. Cell.

[20] Wang H., Jurado K.A., Wu X., Shun M.C., Li X., Ferris A.L., Smith S.J., Patel P.A., Fuchs J.R., Cherepanov P. (2012). HRP2 determines the efficiency and specificity of HIV-1 integration in LEDGF/p75 knockout cells but does not contribute to the antiviral activity of a potent LEDGF/p75-binding site integrase inhibitor. Nucleic Acids Res..

[21] Schrijvers R., Vets S., De Rijck J., Malani N., Bushman F.D., Debyser Z., Gijsbers R. (2012). HRP-2 determines HIV-1 integration site selection in LEDGF/p75 depleted cells. Retrovirology.

[22] Wu H., Zeng H., Lam R., Tempel W., Amaya M.F., Xu C., Dombrovski L., Qiu W., Wang Y., Min J. (2011). Structural and Histone Binding Ability Characterizations of Human PWWP Domains. PLoS ONE.

[23] LeRoy G., Oksuz O., Descostes N., Aoi Y., Ganai R.A., Kara H.O., Yu J.R., Lee C.H., Stafford J., Shilatifard A. (2019). LEDGF and HDGF2 relieve the nucleosome-induced barrier to transcription in differentiated cells. Sci. Adv..

[24] Zhu X., Lan B., Yi X., He C., Dang L., Zhou X., Lu Y., Sun Y., Liu Z., Bai X. (2020). HRP2-DPF3a-BAF complex coordinates histone modification and chromatin remodeling to regulate myogenic gene transcription. Nucleic Acids Res..

[25] Baude A., Aaes T.L., Zhai B., Al-Nakouzi N., Oo H.Z., Daugaard M., Rohde M., Jäättelä M. (2015). Hepatoma-derived growth factor-related protein 2 promotes DNA repair by homologous recombination. Nucleic Acids Res..

[26] Waybright J.M., Clinkscales S.E., Barnash K.D., Budziszewski G.R., Rectenwald J.M., Chiarella A.M., Norris-Drouin J.L., Cholensky S.H., Pearce K.H., Herring L.E. (2021). A Peptidomimetic Ligand Targeting the Chromodomain of MPP8 Reveals HRP2′s Association with the HUSH Complex. ACS Chem. Biol..

[27] Wang J., Zhu X., Dang L., Jiang H., Xie Y., Li X., Guo J., Wang Y., Peng Z., Wang M. (2022). Epigenomic reprogramming via HRP2-MINA dictates response to proteasome inhibitors in multiple myeloma with t(4;14) translocation. J. Clin. Investig..

[28] Kokura K., Sun L., Bedford M.T., Fang J. (2010). Methyl-H3K9-binding protein MPP8 mediates E-cadherin gene silencing and promotes tumour cell motility and invasion. EMBO J..

[29] Dong C., Wu Y., Wang Y., Wang C., Kang T., Rychahou P.G., Chi Y.I., Evers B.M., Zhou B.P. (2013). Interaction with Suv39H1 is Critical for Snail-mediated E-cadherin Repression in Breast Cancer. Oncogene.

[30] Hathaway N.A., Bell O., Hodges C., Miller E.L., Neel D.S., Crabtree G.R. (2012). Dynamics and memory of heterochromatin in living cells. Cell.

[31] Lu D., Foley C.A., Birla S.V., Hepperla A.J., Simon J.M., James L.I., Hathaway N.A. (2022). Bioorthogonal Chemical Epigenetic Modifiers Enable Dose-Dependent CRISPR Targeted Gene Activation in Mammalian Cells. ACS Synth. Biol..

[32] Feldman N., Gerson A., Fang J., Li E., Zhang Y., Shinkai Y., Cedar H., Bergman Y. (2006). G9a-mediated irreversible epigenetic inactivation of Oct-3/4 during early embryogenesis. Nat. Cell Biol..

[33] Lu P., Hostager B.S., Rothman P.B., Colgan J.D. (2013). Sedimentation and Immunoprecipitation Assays for Analyzing Complexes that Repress Transcription. Methods Mol. Biol..

[34] Fernandez-Martinez J., LaCava J., Rout M.P. (2016). Density Gradient Ultracentrifugation to Isolate Endogenous Protein Complexes after Affinity Capture. Cold Spring Harb. Protoc..

[35] Moon K.J., Mochizuki K., Zhou M., Jeong H.S., Brady J.N., Ozato K. (2005). The Bromodomain Protein Brd4 Is a Positive Regulatory Component of P-TEFb and Stimulates RNA Polymerase II-Dependent Transcription. Mol. Cell.

[36] Prigozhin D.M., Douse C.H., Farleigh L.E., Albecka A., Tchasovnikarova I.A., Timms R.T., Oda S.I., Adolf F., Freund S.M.V., Maslen S. (2020). Periphilin self-association underpins epigenetic silencing by the HUSH complex. Nucleic Acids Res..

[37] Bierer B.E., Mattila P.S., Standaert R.F., Herzenberg L.A., Burakoff S.J., Crabtree G., Schreiber S.L. (1990). Two distinct signal transmission pathways in T lymphocytes are inhibited by complexes formed between an immunophilin and either FK506 or rapamycin. Proc. Natl. Acad. Sci. USA.

[38] Sun L., Kokura K., Izumi V., Koomen J.M., Seto E., Chen J., Fang J. (2015). MPP8 and SIRT1 crosstalk in E-cadherin gene silencing and epithelial-mesenchymal transition. EMBO Rep..

[39] Huang Z., Yu P., Tang J. (2020). Characterization of Triple-Negative Breast Cancer MDA-MB-231 Cell Spheroid Model. Onco. Targets Ther..

[40] Izumoto Y., Kuroda T., Harada H., Kishimoto T., Nakamura H. (1997). Hepatoma-Derived Growth Factor Belongs to a Gene Family in Mice Showing Significant Homology in the Amino Terminus. Biochem. Biophys. Res. Commun..

[41] Yang J., Everett A.D. (2007). Hepatoma derived growth factor binds DNA through the N-terminal PWWP domain. BMC Mol. Biol..

[42] Hung Y.L., Lee H.J., Jiang I., Lin S.C., Lo W.C., Lin Y.J., Sue S.C. (2015). The First Residue of the PWWP Motif Modulates HATH Domain Binding, Stability, and Protein-Protein Interaction. Biochemistry.

[43] Lomberk G., Bensi D., Fernandez-Zapico M.E., Urrutia R. (2006). Evidence for the existence of an HP1-mediated subcode within the histone code. Nat. Cell Biol..

[44] Kwon S.H., Florens L., Swanson S.K., Washburn M.P., Abmayr S.M., Workman J.L. (2010). Heterochromatin protein 1 (HP1) connects the FACT histone chaperone complex to the phosphorylated CTD of RNA polymerase II. Genes Dev..

