# Supplementary material for: Characterization of Hepatoma-Derived Growth Factor-Related Protein 2 Interactions with Heterochromatin
Source: Cells. 2023 Jan 14;12(2):325. doi: 10.3390/cells12020325 (PMC9856275; doi:10.3390/cells12020325)
Supplement: Supplementary file 1 [file cells-12-00325-s001.zip › cells-2127733-supplementary.pdf]

Wistner et al., Figure S1

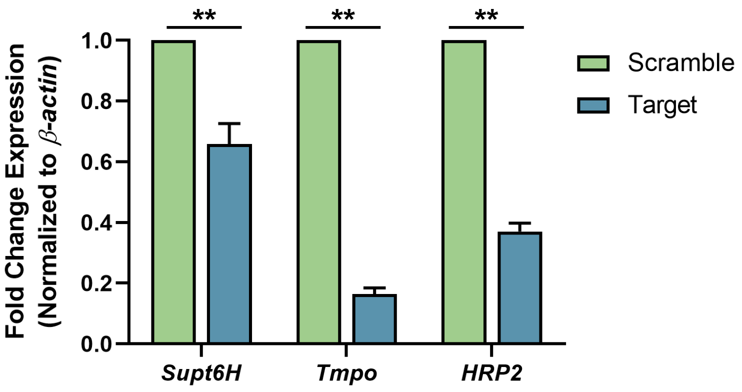

Figure S1: qRT-PCR confirms knockdown of shRNA-targeted genes**.** CiA:mESCs containing inducible shRNA constructs targeted against *Supt6H*, *Tmpo*, and *Hdgfrp2* were induced with 1µg/mL doxycycline for 48 hrs +/- 6 nM rapamycin. Knockdown was confirmed by extracting total RNA for qRT-PCR. Samples were normalized against *β-actin* to determine fold change in expression using comparative *ΔΔ*Ct method. Statistical significance was calculated using an unpaired T-test (n = 3; *p ≤ 0.05, **p ≤ 0.01, ***p ≤ 0.001).

Wistner et al., Figure S2

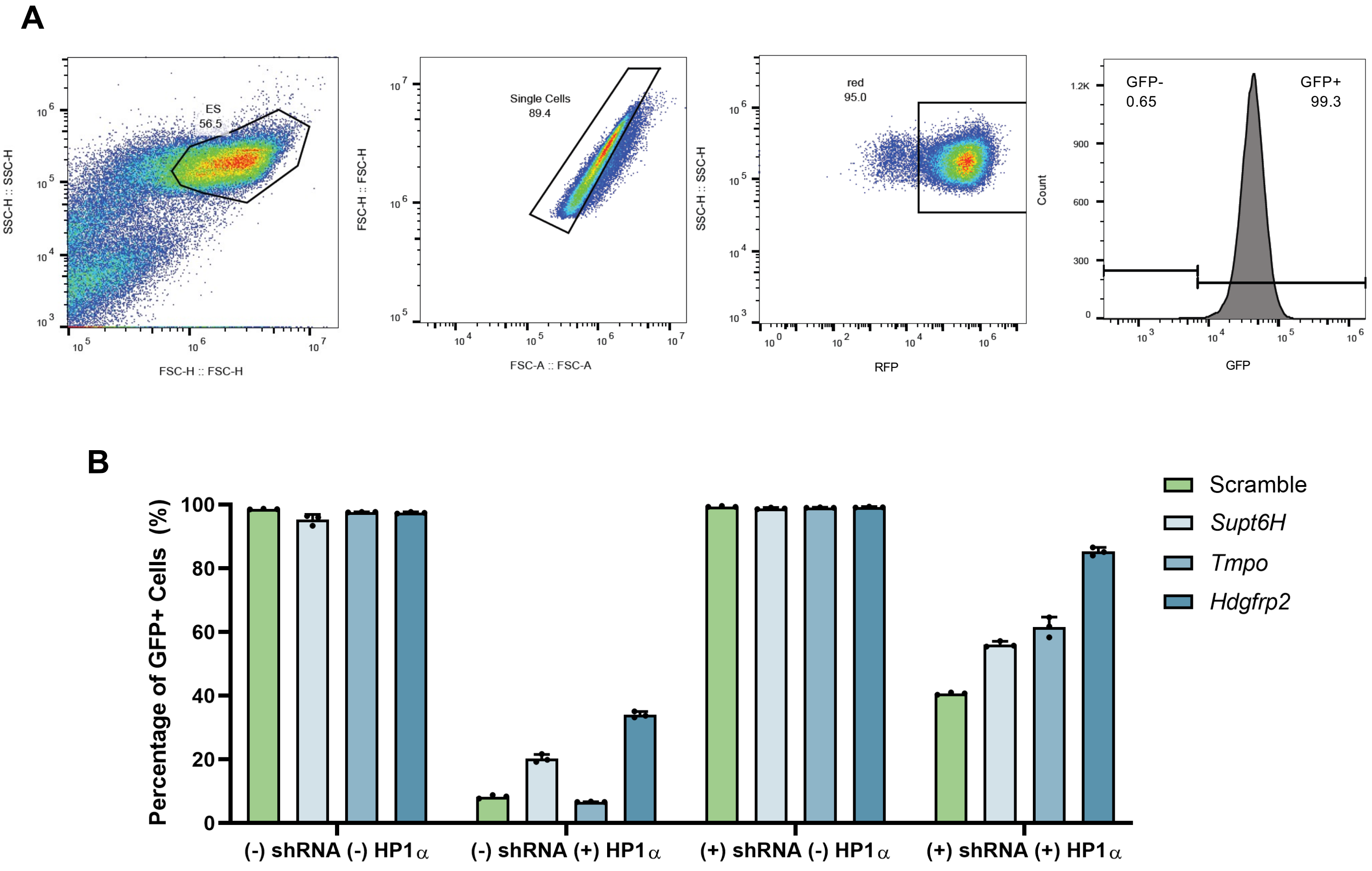

Figure S2: *Supt6H*, *Tmpo*, and *Hdgfrp2* shRNA knockdowns inhibit HP1α-mediated gene silencing to varying degrees**.** (**A**) Representative flow cytometry gating for ES cell, single cell, RFP+, and GFP+ subpopulations. (**B**) CiA:mESCs containing inducible shRNA constructs targeted against *Supt6H*, *Tmpo*, and *Hdgfrp2* were induced with (+) or without (-) 1µg/mL doxycycline (shRNA) for 48 hrs with (+) or without (-) 6 nM rapamycin (HP1α) for 48 hours. Percentage of GFP+ cells following each treatment condition was measured using flow cytometry.

Wistner et al., Figure S3

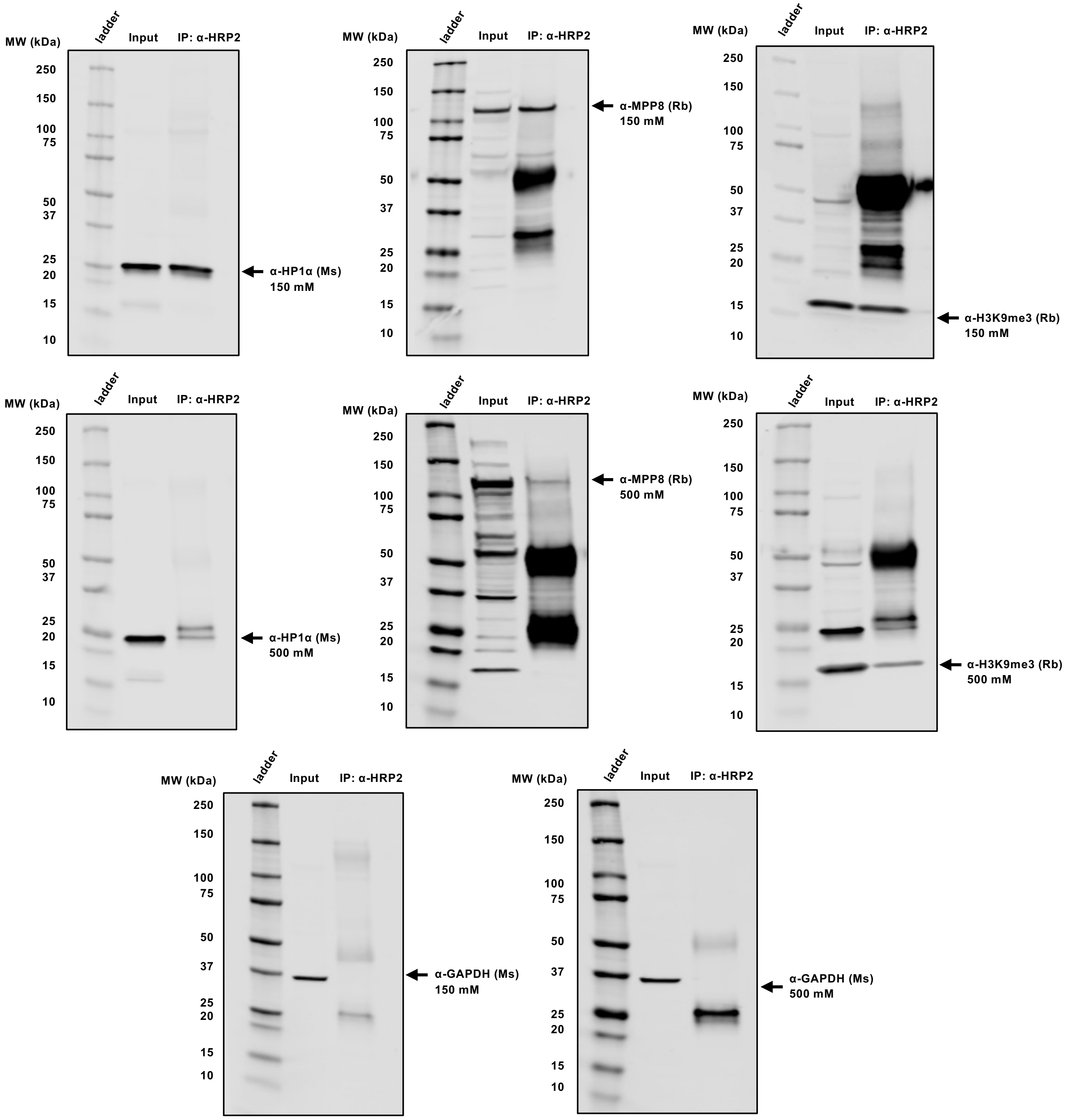

Figure S3: Full immunoblots corresponding to Figure 2. “Ms” indicates mouse primary antibody, “Rb” indicates rabbit primary antibody. High signals at 50 kDa and 25 kDa in IPs using Rb primary antibody are the result of denatured HRP2 Rb antibody used for IP.

Wistner et al., Figure S4

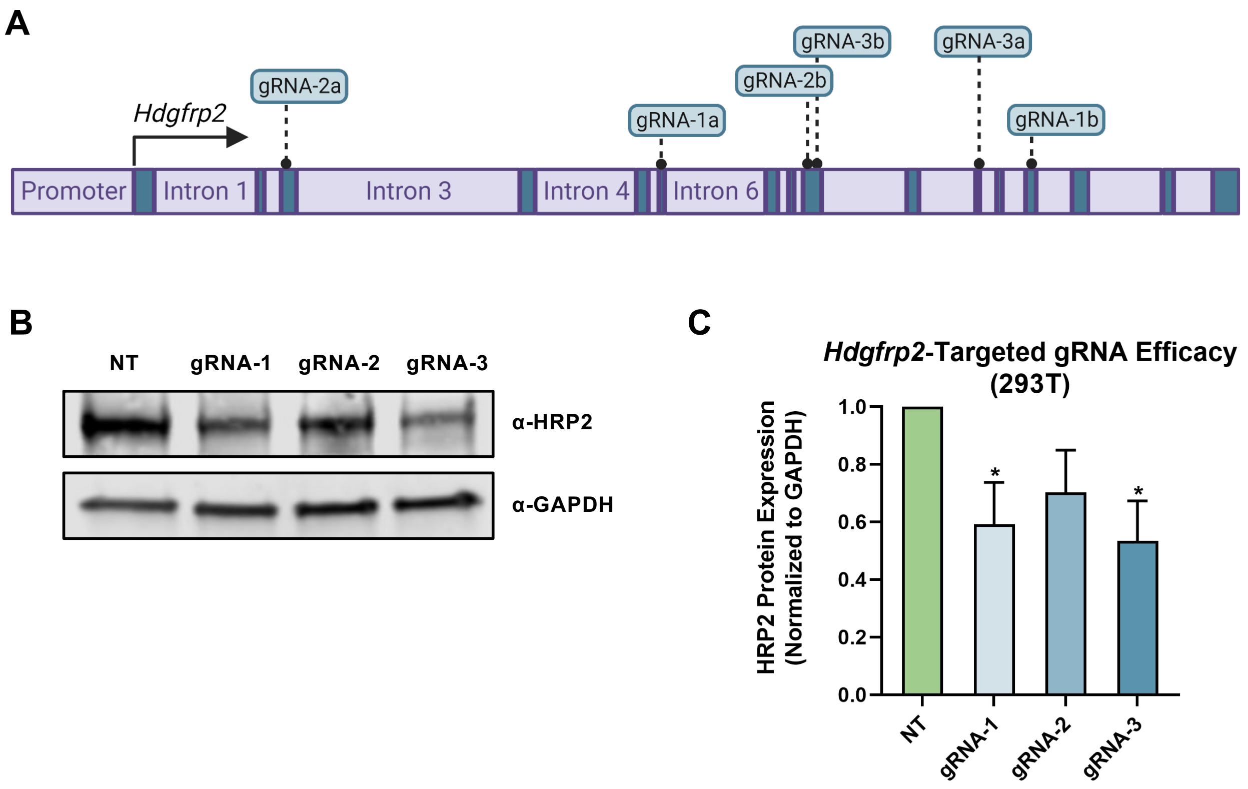

Figure S4: Determination of *Hdgfrp2*-targeted gRNA efficacy. (**A**) Map of CRISPR dual cutter gRNA target sites along *Hdgfrp2* gene. (**B**) Representative immunoblot of whole-cell 293T lysates following transfection with either a non-targeting gRNA (NT) or *Hdgfrp2*-targeted CRISPR dual cutter gRNAs (gRNA-1, gRNA-2, gRNA-3). (**C**) Densitometry quantification of *Hdgfrp2*-targeted gRNA efficacy. Data derived from the average of biological triplicates. Statistical significance was calculated using an unpaired T-test ($n \geq 3$; *$p \leq 0.05$, **$p \leq 0.01$, ***$p \leq 0.001$).

Wistner et al., Figure S5

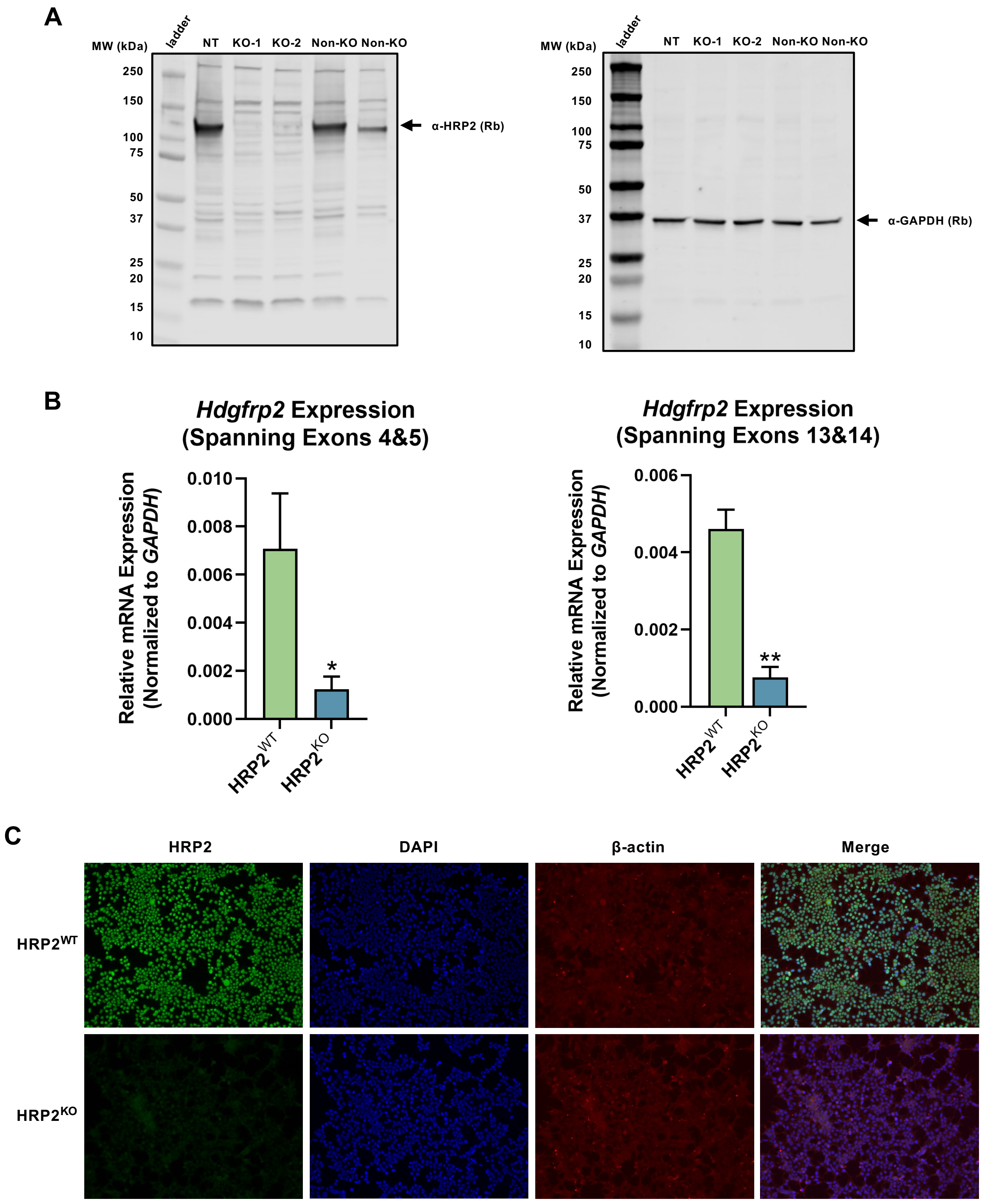

Wistner et al., Figure S5

Figure S5: Validation of *Hdgfrp2*-targeted CRISPR/Cas9 knockout in 293T cells. (**A**) Full immunoblots identifying HRP2 knockout populations, KO-1 and KO-2. Non-KO lanes represent unsuccessful gene knockout colonies. Following validation experiments, all remaining 293T knockout experiments were completed with the KO-1 population, henceforth referred to as $HRP2^{KO}$ (represented in Figure 2A). (**B**) Left: Relative *Hdgfrp2* mRNA expression at a locus spanning exons 4&5, located upstream of the CRISPR dual cutter gRNA-3 cut sites. Right: Relative *Hdgfrp2* mRNA expression at a locus spanning exons 13&14, located downstream of the CRISPR dual cutter gRNA-3 cut sites. Samples were normalized against GAPDH to determine relative expression using comparative $\Delta\Delta$Ct method. Statistical significance was calculated using an unpaired T-test (n = 3; *p ≤ 0.05, **p ≤ 0.01, ***p ≤ 0.001). (**C**) Immunofluorescence microscopy validation of HRP2 knockout. The localizations of antibodies against HRP2 and β-actin were detected using AlexaFluor 488 (green) and AlexaFluor 568 (red), respectively. Cells were co-stained with DAPI (blue) to identify nuclei.

Wistner et al., Figure S6

A

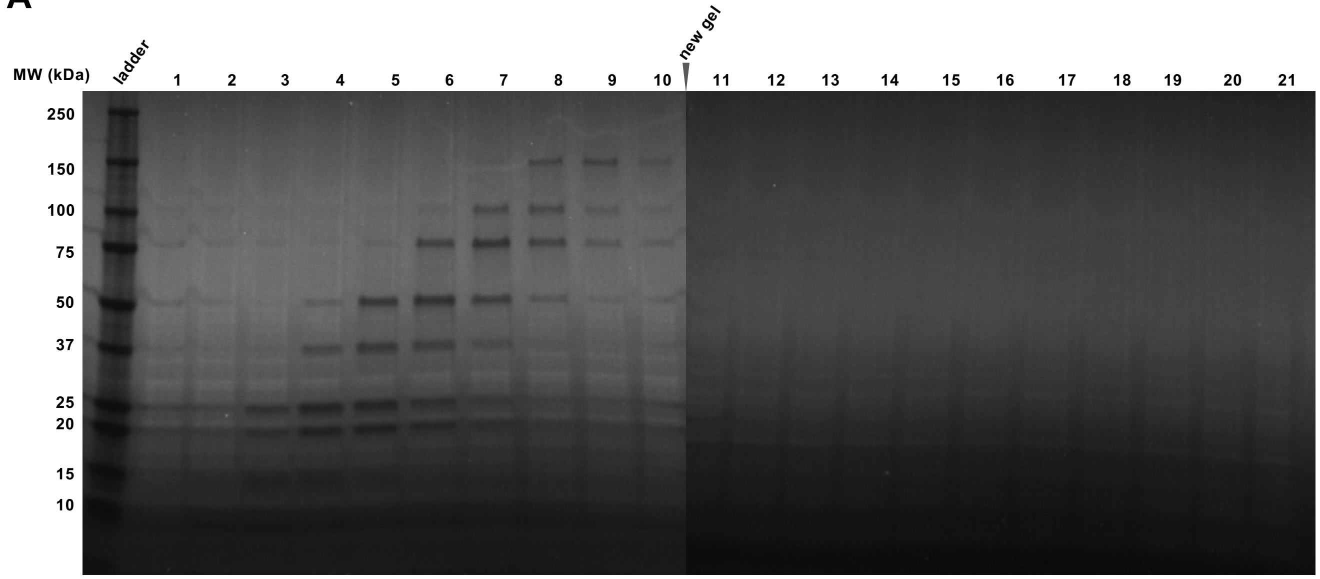

B

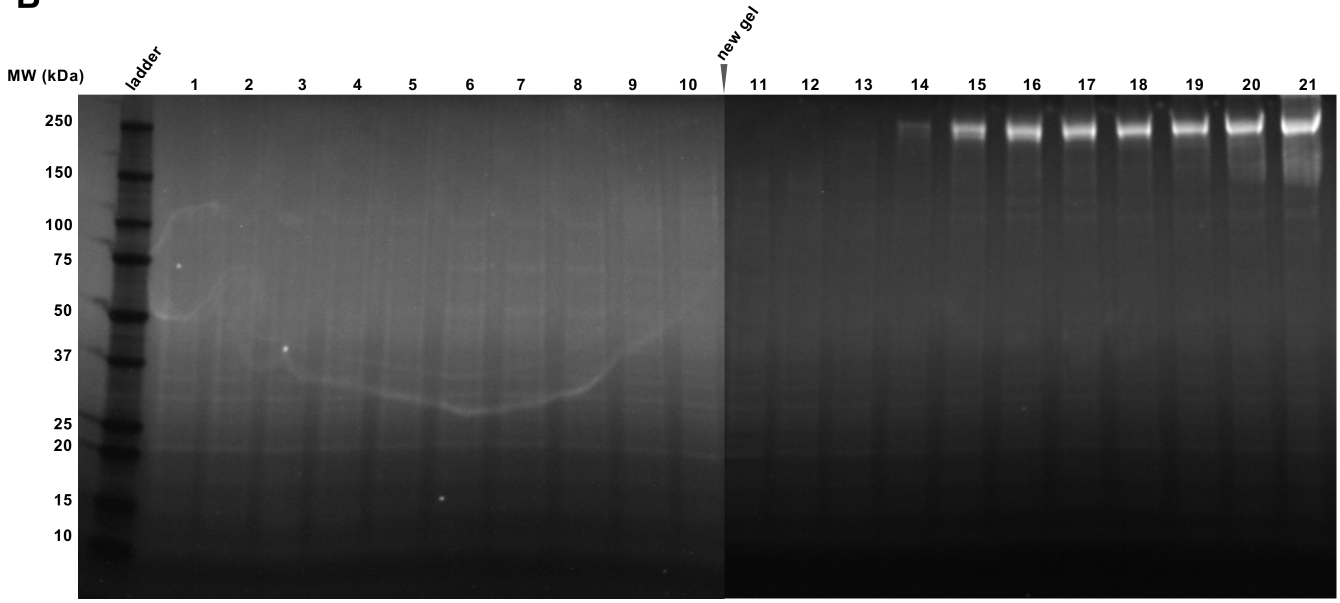

Wistner et al., Figure S6

Figure S7: Sucrose gradient ultracentrifugation and fractionation of protein standards. All standards were run on a 5-20% sucrose gradient. The resulting gradients were fractionated in 500 µl aliquots from lowest density (1) to highest density (21). (**A**) SYPRO Ruby staining of Precision Plus Protein standards after density gradient ultracentrifugation and fractionation. Standards were used to determine the molecular weight ranges of proteins within gradient fractions (fractions 3-5: 25-75 kDa; fractions 7-10: 100-150 kDa). (**B**) SYPRO Ruby staining of thyroglobulin protein standard after density gradient ultracentrifugation and fractionation. Thyroglobulin is a dimeric glycoprotein (660 kDa) and was used to determine the molecular weight ranges of proteins within gradient fractions (fractions 15-21: 330-660 kDa).

Wistner et al., Figure S7

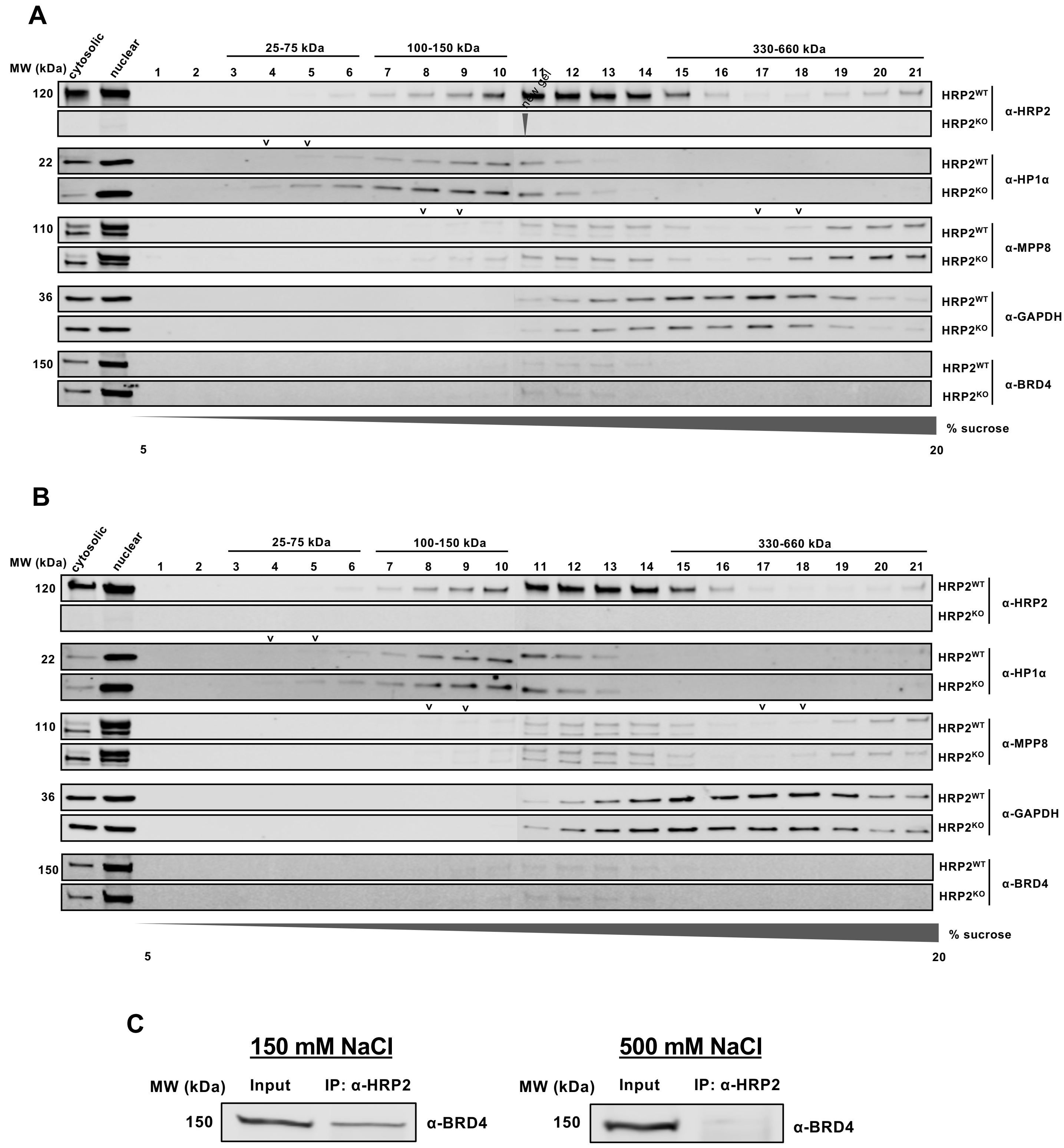

Wistner et al., Figure S7

Figure S7: Biological repeats of sucrose gradient fractions. (**A**) Biological 1: Immunoblot detecting putative molecular complexes associated with HRP2, resulting from a single density gradient ultracentrifugation and fractionation experiment. 1.3 mg of HRP2^{WT} or HRP2^{KO} 293T nuclear lysate was run on a 5-20% sucrose gradient. The resulting gradient was fractionated in 500 µl aliquots from lowest density (1) to highest density (21). Fractions distinguished with "**v**" indicate leftward shifts of proteins to lower density fractions in the absence of HRP2 expression compared to wildtype conditions. Of note, the BRD4 control was excluded from the main text due to limited detection of bands. (**B**) Biological 2: Additional immunoblot detecting putative molecular complexes associated with HRP2, resulting from a second density gradient ultracentrifugation and fractionation experiment. (**C**) Immunoblots verifying that HRP2 does not interact with BRD4, an epigenetic activator, at 500 mM NaCl.

Wistner et al., Figure S8

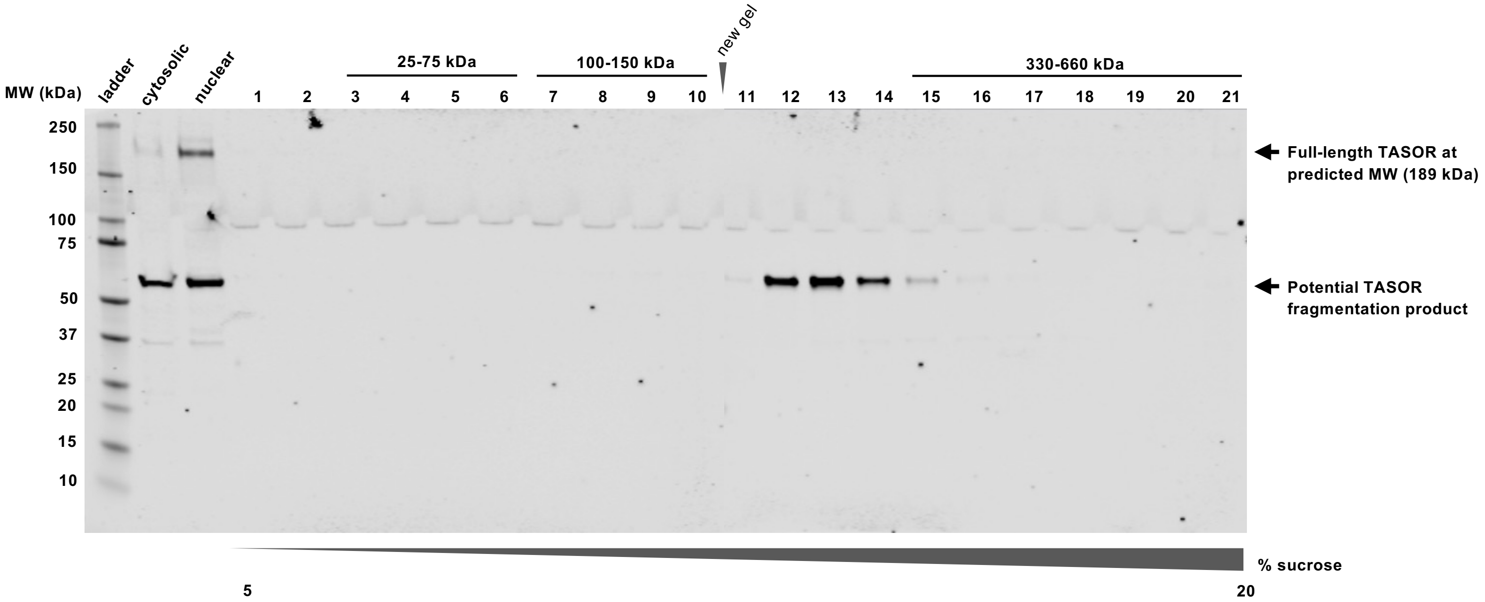

Figure S8: Incomplete capture of TASOR migration within sucrose gradient fractions. Full immunoblot of HRP2^{WT} 293T sucrose gradient fractions probed with an anti-TASOR antibody. 1.3 mg of nuclear lysate was run on a 5-20% sucrose gradient. The resulting gradient was fractionated in 500 µl aliquots from lowest density (1) to highest density (21). Arrows indicate full-length TASOR present in cytoplasmic and nuclear lysate inputs, as well as a potential TASOR fragmentation product within the gradient.

Wistner et al., Figure S9

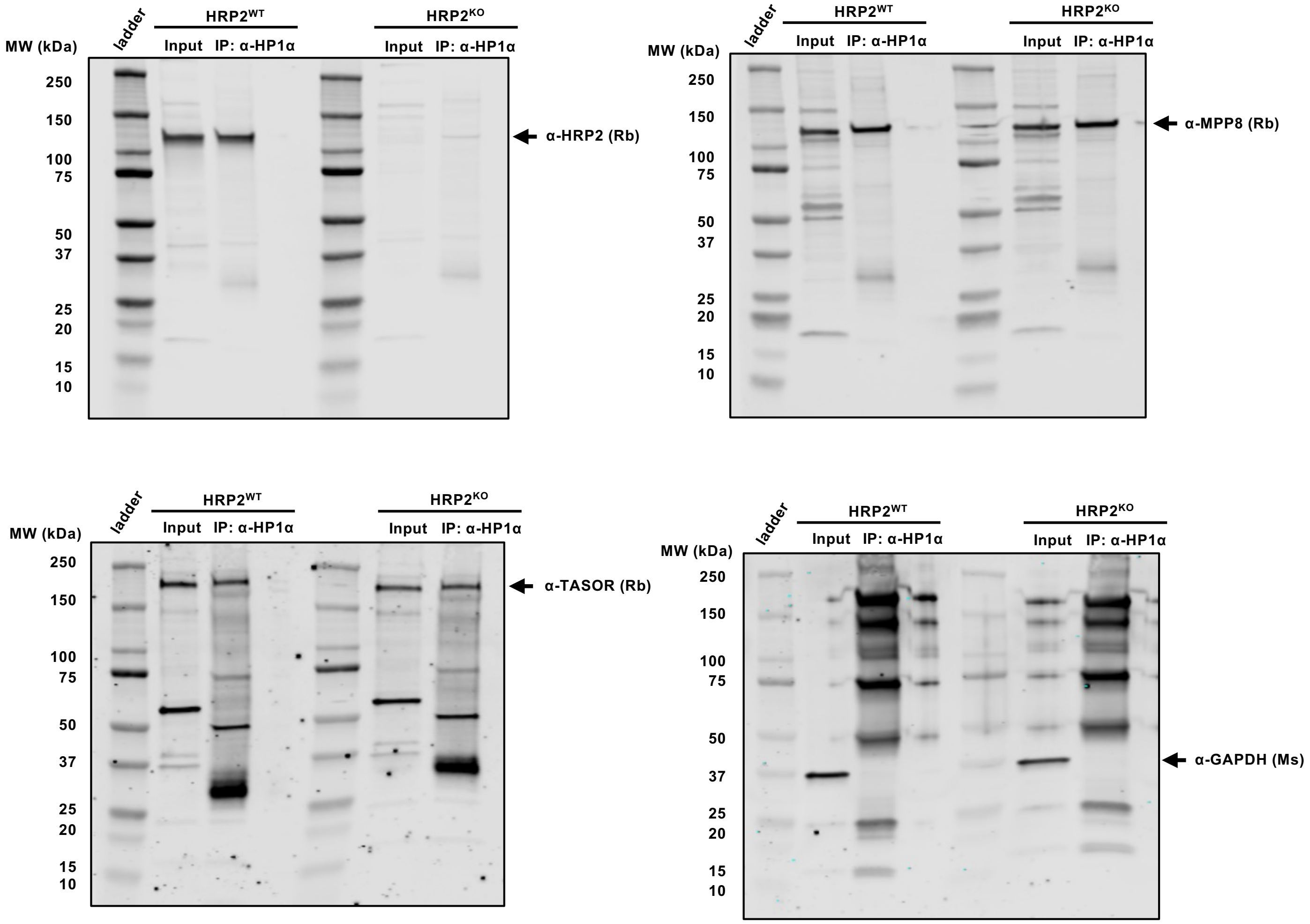

Figure S9: Full immunoblots corresponding to Figure 3D. “Ms” indicates mouse primary antibody, “Rb” indicates rabbit primary antibody.

Wistner et al., Figure S10

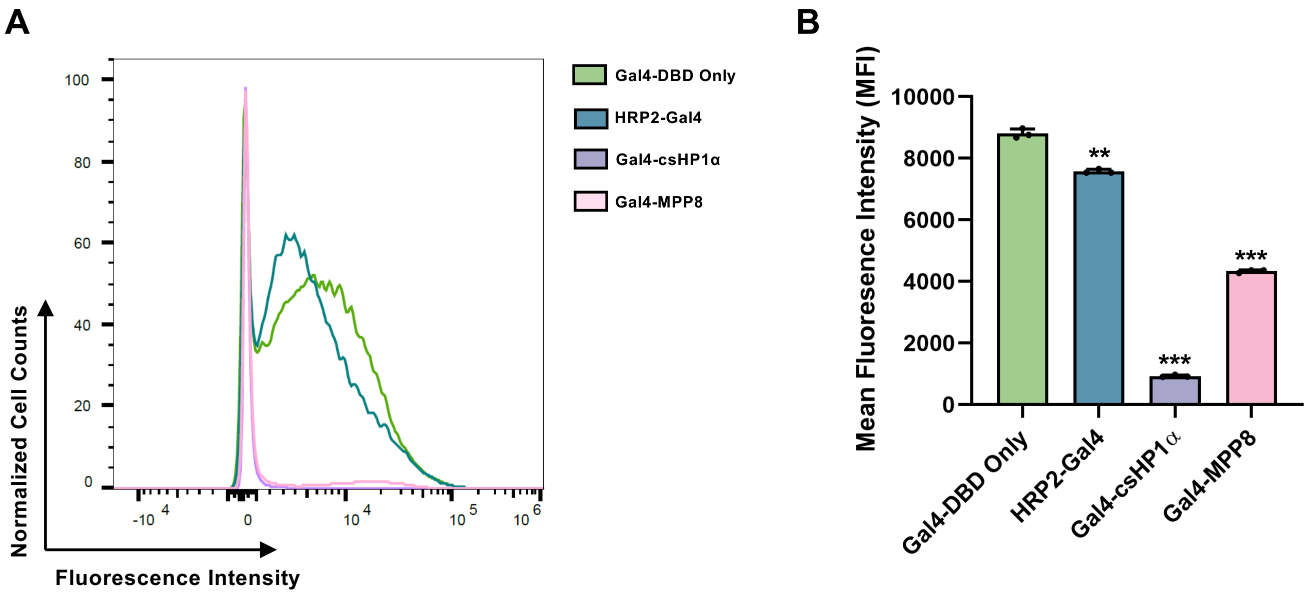

Figure S10: HRP2 direct recruitment induces slight but not complete gene silencing. (**A**) Representative flow cytometry histogram measuring fluorescence intensity of CiA:293T cells following stable recruitment of Gal4-DBD Only (green), HRP2-Gal4 (blue), Gal4-csHP1α (purple), or Gal4-MPP8 (pink) to a GFP reporter locus. (**B**) Mean fluorescence intensity values of CiA:293T cells represented in A. Statistical significance was calculated using an unpaired T-test (n = 3; $*p \leq 0.05$, $**p \leq 0.01$, $***p \leq 0.001$).

Wistner et al., Figure S11

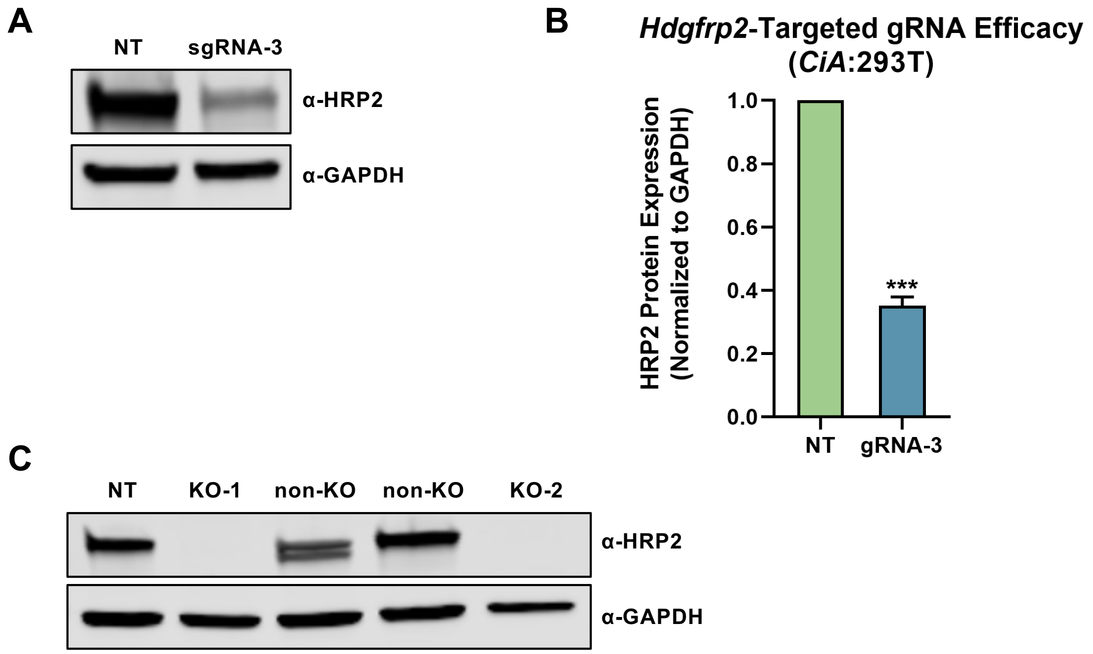

Figure S11: Validation of *Hdgfrp2*-targeted CRISPR/Cas9 knockout in CiA:293T cells. (**A**) Representative immunoblot of whole-cell CiA:293T lysates following transfection with either a non-targeting gRNA (NT) or *Hdgfrp2*-targeted CRISPR dual cutter gRNA-3. (**B**) Densitometry quantification of *Hdgfrp2*-targeted gRNA efficacy. Data derived from the average of biological triplicates. (**C**) Immunoblot identifying HRP2 knockout populations, KO-1 and KO-2. Non-KO lanes represent unsuccessful gene knockout colonies. Following validation experiments, all remaining CiA:293T knockout experiments were completed with the KO-2 population, henceforth referred to as $HRP2^{KO}$. Statistical significance was calculated using a paired T-test Statistical significance was calculated using an unpaired T-test (n = 3; *p ≤ 0.05, **p ≤ 0.01, ***p ≤ 0.001).

Wistner et al., Figure S12

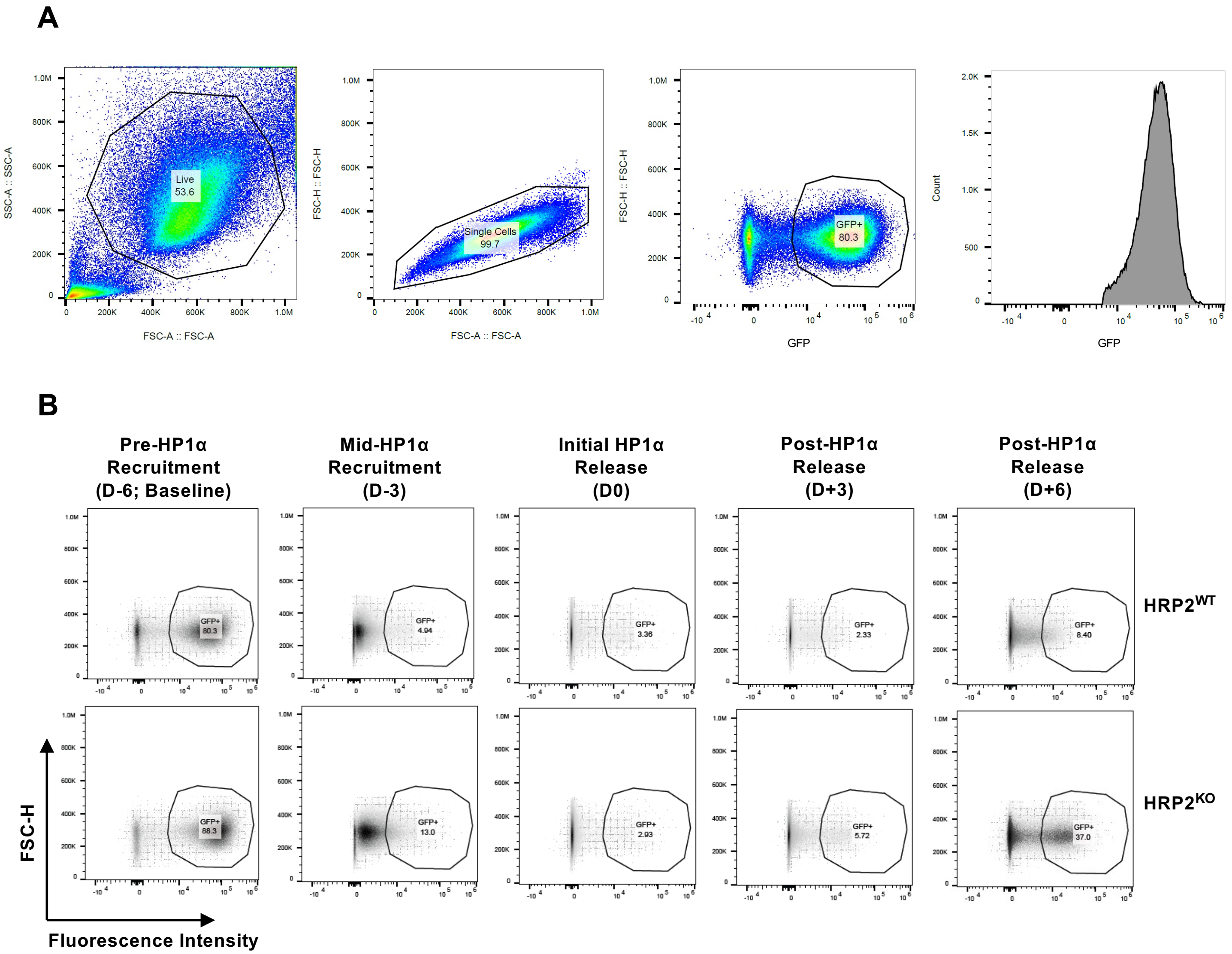

Figure S12: Flow cytometry gating used to compare $HRP2^{WT}$ and $HRP2^{KO}$ GFP+ populations over time. (**A**) Representative flow cytometry gating for live CiA:293T cell, single cell, and GFP+ subpopulations. (**B**) Representative flow cytometry gating used to determine starting percentage of GFP+ cells (baseline) used to calculate relative percentages of GFP+ cells at each time point in Figure 4. Time course was performed in biological triplicate.

Wistner et al., Figure S13

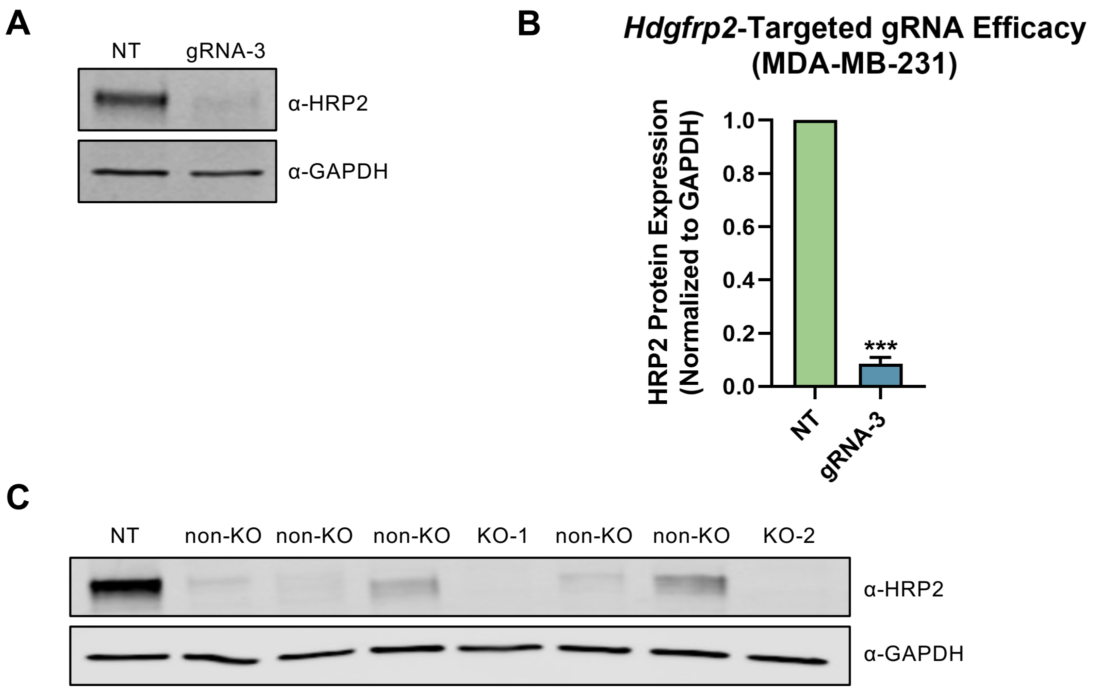

Figure S13: Validation of *Hdgfrp2*-targeted CRISPR/Cas9 knockout in MDA-MB-231 cells. (**A**) Representative immunoblot of whole-cell MDA-MB-231 lysates following transfection with either a non-targeting gRNA (NT) or *Hdgfrp2*-targeted CRISPR dual cutter gRNA-3. (**B**) Densitometry quantification of *Hdgfrp2*-targeted gRNA efficacy. Data derived from the average of biological triplicates. (**B**) Immunoblot identifying HRP2 knockout populations, KO-1 (HRP2^{KO-1}) and KO-2 (HRP2^{KO-2}). Non-KO lanes represent unsuccessful gene knockout colonies. Statistical significance was calculated using a paired T-test Statistical significance was calculated using an unpaired T-test (n = 3; *$p \leq 0.05$, **$p \leq 0.01$, ***$p \leq 0.001$).

Wistner et al., Figure S14

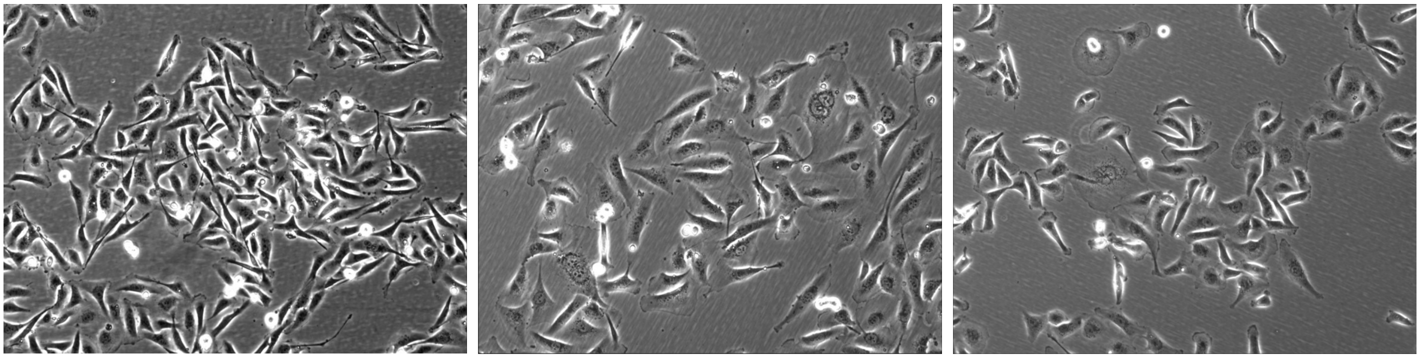

HRP2^{WT} HRP2^{KO-1} HRP2^{KO-2}

Figure S14: HRP2 knockout induces changes in MDA-MB-231 cellular morphology, including increased size, loss of membrane boundaries, enlarged nuclei, and increased granularity.

Table S1: shRNA Sequences. Format: 5' shRNA hairpin, target sequence, hairpin loop, reverse target sequence, shRNA hairpin 3'. Mouse sequence indicated by "*m*."

Gene	Sequence (5' → 3')
Scramble	AAGGTATATtgctgttgacagtgagcgATCTCGCTTGGGCGAGAGTAAGtagtgaagccacagatgtaCTTACTCTCGCCCAAGCGAGAGtgcctactgcctcgga
mSupt6H	AAGGTATATtgctgttgacagtgagcgCAGCACTGACTCATACATTGAAGTTCTTGtagtgaagccacagatgtaCAAGAACTTCAATGTATGAGTCAGTGCTGtgcctactgcctcgga
mTmpo	AAGGTATATtgctgttgacagtgagcgCCTTCGGTCCTGACCAAAGACAAGTTGAAtagtgaagccacagatgtaTTCAACTTGTCTTTGGTCAGGACCGAAGGtgcctactgcctcgga
mHdgfrp2	AAGGTATATtgctgttgacagtgagcgAAGTAGACCGCATCAGTGAATGGAAGAGAtagtgaagccacagatgtaTCTCTTCCATTCACTGATGCGGTCTACTTtgcctactgcctcgga

Table S2: RNA qRT-PCR Primer Sequences. Mouse sequence indicated by "*m*," human sequence indicted by "*h*."

Gene	Forward Primer (5' → 3')	Reverse Primer (5' → 3')
mSupt6H	GGACCGAAAGAAATTAGAGGA	CAGGCACAGATGAAGTAAGG
mTmpo	CCATTGTGGGAACAACCAG	TAGAGGATCTCGATTCAGTTC C
mHdgfrp2	TGGAGAGCGATTCTGACTC	AGACTGACACCTTCAAGACTG
mβ-actin	CTCCTATGTGGGTGACGAG	TCTCAAACATGATCTGGGTC
hHdgfrp2 (Exons 4&5)	GAGAGCGACTCAGACTCAG	TCGAGACCGACATCTTTAGC
hHdgfrp2 (Exons 13&14)	CACCTTGAAGAAGATTCGCC	GACTTGAGCCGGGTATAGAC
hE-cadherin	AGAGACTGGGTTATTCCTCC	GGATTTGATCTGAACCAGGT
hN-cadherin	CATCCCTCCAATCAACTTGC	TTATCTCTATCAGACCTGATC CTG
hGAPDH	CAATGACCCCTTCATTGACC	TTGATTTTGGAGGGATCTCG

Table S3: ChIP Primer Sequences. TSS = distance from transcriptional start site (in bp).

Gene	Forward Primer (5' → 3')	Reverse Primer (5' → 3')
E-cadherin (TSS -127)	CTCAGCCAATCAGCGGTACG	GCGGGCTGGAGTCTGAAC
N-cadherin (TSS -135)	ACACGCTCTCCCTCCCTGTT	GCCAATGGAGAGCGAGCTGA
